# Climate change provides opportunities for the cultivation of *Coffea arabica* in China, an integrated model analysis based on biomod2

**DOI:** 10.3389/fpls.2026.1806108

**Published:** 2026-03-20

**Authors:** Tiantian Chen, Zhiwei Ding, Liudan Zhang, Li Gong, Song Qing, Xuejun Li, Zhenjiang Lv

**Affiliations:** 1 College of Tropical Crops, Yunnan Agricultural University, Puer, China; 2 Yunnan Key Laboratory of Coffee, Yunnan Agricultural University, Puer, China

**Keywords:** biomod2, climate change, *Coffea arabica*, optimized model, suitable distribution region

## Abstract

**Introduction:**

Global warming is reshaping species distribution patterns.

**Methods:**

Based on climatic, topographic and soil data, the *Coffea arabica* planting regions with different levels of ecological suitability in different periods and the environmental factors that have the largest impact on ecological suitability were simulated using the Biomod2 model to clarify the influence of climate change on the suitable habitats of C. arabica in China.

**Results:**

The results revealed that the temperature seasonality (Bio4), mean temperature of the coldest quarter (Bio11), precipitation of the warmest quarter (Bio18) and elevation (Elev) were the main environmental factors affecting the distribution of *C. arabica*. Under current climatic conditions, the suitable habitats of C. arabica are primarily distributed across Yunnan, Hainan and Guangxi Provinces, with southwestern Yunnan identified as a core highly suitable area. Under future climates, the total area of suitable habitats for *C. arabica* will generally expand, with habitat size positively correlated with temperature stress intensity. Specifically, under the SSP5-8.5 scenario, the suitable habitat area will reach 184.29 × 10^4^ km^2^, tripling the current extent, and may expand toward higher latitudes.

**Discussion:**

Climate warming had increased the area of suitable regions for *C. arabica* in China, which provides a theoretical basis for the introduction, cultivation of *C. arabica*, as well as the planning and management of plateau characteristic agricultural production.

## 1 Introduction

Against the background of global warming, it is climate change that exerts impacts on the function and structure of ecosystems, species diversity, and population genetic diversity (Pio et al., 2014; Ambrósio et al., 2024). As documented in reports released by the Intergovernmental Panel on Climate Change (IPCC), global warming driven by greenhouse gas emissions has already caused 1.09°C increases in global temperatures compared to pre-industrial levels. Over the next two decades, the global average temperature is projected to hit or even surpass the 1.5°C threshold relative to pre-industrial benchmarks, whereas the magnitude of warming expected by the end of the 21st century is estimated to fall within the interval of 1.0°C to 5.7°C (Liu et al., 2019; Allan et al., 2023). Global warming induces a reduction in crop yields (Hasegawa et al., 2021), which not only poses a severe threat to global food security but also undermines the process of sustainable development. Concurrently, species are compelled to undergo adaptive adjustments to cope with a suite of extreme weather events, including the extension of frost-free periods, the occurrence of seasonal droughts, the incidence of heatwaves, and alterations in precipitation patterns (Kurpis et al., 2019; Gilani et al., 2020). As a key environmental factor, climate indirectly modifies plant reproduction, diversity, and dispersal patterns by influencing plant community composition, species distribution, and ecophysiological processes (Wang et al., 2016; Zhao et al., 2024). Future climate change is projected to alter the climatically suitable habitats for plants, posing a severe threat to species diversity (Shen and Wang, 2013; Tian and Jiang, 2015). Plant geographical distribution will shift in response to climate change (Zhang et al., 2019; Kong et al., 2025). Outcomes of a newly published study suggested that the suitability of *C. arabica* habitats in Yunnan Province is collectively shaped by climatic, soil, and topographic factors in the future (Li et al., 2024). *C. arabica* in low-latitude and low-altitude areas is projected to be significantly impacted by climate change. A growing body of prior studies has confirmed that climate change exerts negative influences on coffee cultivation in primary coffee producing regions such as Brazil, Ethiopia, and India (Adane and Bewket, 2021; Byrareddy et al., 2024). Elucidating the impacts of climate change on the geographical distribution patterns of *C. arabica* in China and its adaptive mechanisms to climate change not only provides key theoretical support for ensuring the ecologically sustainable development of China’s coffee industry and optimizing the layout of plateau-specific agriculture, but also is of great significance for maintaining the stability of regional agroecosystems and promoting the high-quality development of the coffee industry (Pramanik et al., 2018; Kakpo et al., 2021).

Species distribution models (SDMs) refer to a tool that utilizes known distribution locations of species and relevant environmental data, employs methods derived from mathematical statistics and machine learning theories, and predicts the distribution trends and diffusion patterns of species (Cai et al., 2023). It play a key role in exploring the interactions between species and the environment, as well as in predicting species distributions (Kearney et al., 2010). Currently, maximum entropy (MaxEnt) models, HABITAT models, and BIOCLIM models are recognized as the most commonly utilized species distribution models in ecological research (Latimer et al., 2006; Zhang et al., 2024). Driven by discrepancies in theoretical principles and algorithmic designs, each model is characterized by specific advantages and limitations, while its performance stability can be compromised by changes in the input data used for modeling (Thuiller, 2003; Wiens et al., 2009). To improve prediction accuracy, Biomod, a modeling tool built on the R software, has gained extensive recognition and found widespread application since its release (Thuiller et al., 2009). As its successor, Biomod2 also operates within the R platform. Beyond enabling the optimization and constraint of parameters across multiple models, it further adopts an Ensemble Model (EM) strategy (Dormann et al., 2018). Currently, the effects of environmental factors on plants have been examined by scholars through the application of these multiple or integrated models (Gan et al., 2025). For example, Wang et al. examined how climate change might affect the distribution of *Rubia cordifolia* across China and determined the pivotal environmental drivers of the species habitat suitability. The findings indicated that the suitable habitats of *Rubia cordifolia* will either contract inward or expand outward along existing habitats in the future, with precipitation of the wettest month being the key variable regulating its distribution (Wang et al., 2023). Wen et al. used the Biomod2 ensemble model and chemical analyses to forecast the current and future distribution of *Leonurus japonicus Houtt*. under climate change, and pinpointed its critical environmental determinants. The findings revealed that precipitation of the wettest month, mean temperature of the wettest quarter, and mean temperature of the warmest quarter are critical factors influencing the habitat suitability of *Leonurus japonicus Houtt.*, with its suitable habitats exhibiting a distinct northward migration trend (Wen et al., 2025). Biomod2 has been increasingly applied to investigating the response mechanisms of various endangered species, invasive species, and economically valuable taxa to environmental variables, and it plays an indispensable role in the fields of species conservation, planning, and management (Fang et al., 2021; Xu et al., 2021).

Coffee (*Coffea* spp.), perennial evergreen dicotyledonous plants belonging to the Rubiaceae family, are among the most widely distributed and valuable crops worldwide (FAO, 2022), and the most consumed non-alcoholic beverages (Wang et al., 2022). In Asia, China ranks as the fourth largest coffee exporter after Vietnam, Indonesia, and India. Currently, *C. arabica* is the most extensively cultivated coffee species worldwide, representing roughly 80% of the global coffee cultivation area (ICO, 2022). In China, the distribution of *C. arabica* is mainly concentrated in Yunnan Province, where coffee production accounts for 98.8% of the national total, with excellent quality (Wang et al., 2022). However, *C. arabica* is particularly sensitive to climate (Davis et al., 2012), and coffee trees are susceptible to agrometeorological disasters such as cold and hot temperatures, suitable climate conditions are crucial for ensuring their normal growth (Davis et al., 2019). When the daily minimum temperature drops below 0°C, coffee trees may suffer from freezing damage, leading to death and leaf wilting or discoloration, excessively high daily maximum temperatures are unfavorable for flowering and fruiting (Chen, 2019). Precipitation is also an important factor for coffee growth, excessive rainfall can cause flower abscission and fruit injury, while insufficient rainfall may result in drought and reduced yields (Läderach et al., 2017). The global area suitable for coffee cultivation will be reduced by around 50% as a consequence of climate change stemming from high-level emissions (Bunn et al., 2015). Accordingly, some scholars in China has investigated the effects of climate change on the suitable habitats of *C. arabica.* For instance, Zhu et al. utilized the MaxEnt model to forecast the suitable habitats of *C. arabica* in Yunnan Province under current and future climate regimes. The results indicated that future climate change will lead to a reduction in the total area of suitable habitats for *C. arabica* in Yunnan, with the centroid of the total suitable habitats shifting toward regions with higher altitudes and latitudes (Zhu et al., 2021). Zhang et al. used the AHP-GIS and MaxEnt models to predict *C. arabica* potential distribution in Yunnan. The results demonstrated that under future climate scenarios, climatic factors exert the most prominent influence on the suitable habitats of *C. arabica*. Meanwhile, the overall suitable habitats exhibit a distinct northward migration trend toward higher altitudes and latitudes, with the newly expanded areas predominantly concentrated in western, southwestern, and southeastern Yunnan Province (Zhang et al., 2021). Although most studies have projected the impacts of climate change on *C.arabica*, the majority rely on a single modeling approach with the research scope confined to Yunnan Province. In contrast, the distribution dynamics of environmentally suitable areas for *C. arabica* cultivation across the entire China, as predicted by ensemble modeling methods, remain unclear to date. Therefore, researching the impact of climate change on the geographical distribution of *C. arabica* is helpful for understanding its geographical distribution pattern and providing a scientific basis for the rational development and utilization of *C. arabica*. Theoretical support for the research, development and utilization of *C. arabica* germplasm resources can be derived from an in-depth grasp of the species climatic adaptability and the implementation of corresponding adaptive adjustments.

Based on species occurrence data and climate, topography, soil data, the ensemble model in Biomod2 was employed to predict the potential geographical distribution of *C. arabica*, thereby revealing its response patterns to climate change across different periods in a scientific manner. The aims of this study are as follows: (i) to clarify the geographical distribution characteristics of *C. arabica* under current climatic conditions; (ii) to identify the key environmental factors influencing the geographical distribution of *C. arabica*; (iii) to predict the potential geographical distribution and spatial change patterns of suitable habitats for *C. arabica* under future climate change scenarios. It is expected that the results of this study will provide scientific support for the development and sustainable utilization of *C. arabica* germplasm resources.

## 2 Material and methods

### 2.1 Collection and screening of sample data

**Figure 1:**
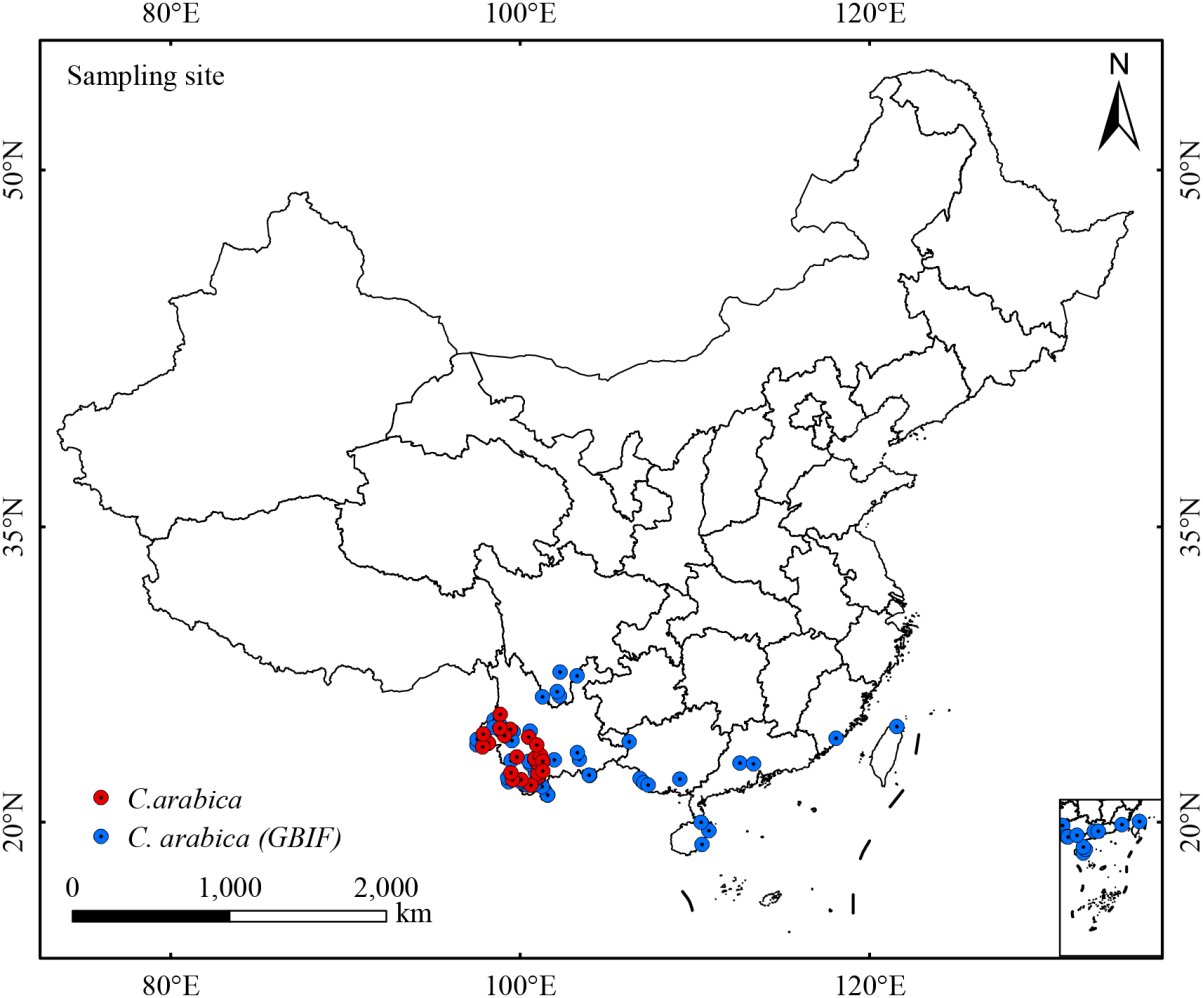
Spatial distribution of *C. arabica* occurrence recorded in China. The base map is produced based on the standard map of China published by the Ministry of Natural Resources, with the review number GS (2024) 0650. The same below.

Relevant data were retrieved from the Global Biodiversity Information Facility (GBIF, http://www.gbif.org) database, and field surveys targeting *C. arabica* were conducted in Yunnan Province from December 2024 to January 2025, yielding a total of 1948 geo-referenced occurrence records of the species. First, data without specific longitude and latitude were removed. First, records lacking precise geographic coordinates and specimen-based entries were excluded, with only cultivation-associated data retained. The filtered dataset was then imported into ArcGIS v10.8, where the SDMToolbox extension was applied to remove redundant occurrence records within a 1 km radius of each focal site, thereby mitigating spatial sample autocorrelation. Ultimately, 59 occurrence points covering the entire distribution range of *C. arabica* were obtained (**Figure 1**).

### 2.2 Processing of environmental variables

Current and future climate and topographic data were retrieved from WorldClim (http://worldclim.org) at a 30″ resolution. For future climate variables, the BCC-CSM2-MR model from the Coupled Model Intercomparison Project Phase 6 (CMIP6) was adopted, incorporating four combined scenarios of Shared Socioeconomic Pathways and Representative Concentration Pathways (SSP126, SSP245, SSP370, SSP585) for the 2030s, 2050s, 2070s, and 2090s. This model exhibits favorable performance in reproducing the climatological mean states and interannual variability of air temperature, precipitation, and monsoon circulations over China and East Asia, with particularly outstanding skill in simulating temperature conditions across the mountainous regions of the southwestern plateaus. The raw climate data for each period included 19 bioclimatic variables. Soil factor data were acquired from the FAO Harmonized World Soil Database (HWSD, http://www.fao.org/faostat/en/#data.). Topographic and soil variables are not expected to undergo significant changes in the coming decades; thus, the same fixed topographic and soil data were used for both current and future periods in this study (Debella-Gilo and Etzelmüller, 2009). The resampling tool in ArcToolbox of ArcGIS 10.8 was employed to harmonize the coordinate systems and geographic extents of all environmental variables. The contribution rates of environmental factors were quantified using R packages, followed by Pearson correlation analysis of all environmental variables implemented in ENMTools. Environmental factors with a contribution rate ≤ 0.5 were excluded; for pairs of factors with an absolute correlation coefficient |r| ≥ 0.8, only the variable with the higher contribution rate was retained.

### 2.3 Model parameterization

Based on the Biomod2 package integrated in R (v4.4.1), 12 species distribution models were established using *C. arabica* occurrence data and screened environmental factors. The selected models included Artificial Neural Network model (ANN), Classification Tree Analysis (CAT), Flexible Discriminant Analysis (FDA), Generalized Additive Model (GAM), Generalized Boosted Regression Models (GBM), Generalized Linear Model (GLM), Maximum Entropy model (MaxEnt), Multivariate Adaptive Regression Splines (MARS), Maximum Picking Neural Network (MaxNet), Random Forest model (RF), Extreme Gradient Boosting model (XGBOOST), and Surface Range Envelope (SRE) (Gong et al., 2020; Huang et al., 2023). For the MaxEnt model, two core parameters, regularization multiplier (RM) and feature combination (FC) were optimized *a priori* via the ENMeval 2.0 package (Kass et al., 2021). Both pre-optimized MaxEnt models and post-optimized MaxNet models were constructed. The RM values were set in the range [0.5, 4] at intervals of 0.5, resulting in 8 values. For FC, combinations including L, LQ, LQH, H, LQHP, and LQHPT were used (where L = linear, Q = quadratic, H = hinge, P = product, T = threshold), resulting in 48 parameter combinations for ENMeval validation. Models with the lowest AICc (delta.AICc = 0) and high AUC values (AUC>0.9) performed best (Zhao et al., 2021), while models with delta.AICc<2 were also reliable (Phillips et al., 2017). The optimized parameters obtained were, RM = 0.5 and FC = LQ. Except for MaxEnt, all other algorithms used the default settings in Biomod2. In the modeling process, 75% of the 59 occurrence samples were randomly selected as training data, and the remaining 25% as test data, with each model run 10 times. Furthermore, for the purpose of enhancing the consistency between model outputs and actual distribution patterns and reducing spatial deviation, 1000 pseudo-absence points were randomly generated, and the entire process was repeated twice. The prevalence was set to 0.5 to ensure the weighted sum of presence points and pseudo-absence points was equal. Model performance was assessed based on two metrics, Receiver Operating Characteristic (ROC) and True Skill Statistics (TSS). Widely recognized as one of the most valid SDM evaluation indicators, AUC is free from specific diagnostic threshold constraints and exhibits low sensitivity to species occurrence frequency fluctuations, which ensures reliable evaluation outcomes. AUC values (0.5-1) correlate positively with model predictive accuracy (Muscarella et al., 2014). As a model evaluation metric, TSS retains the merits of the Kappa algorithm while overcoming its limitation in capturing unimodal curve responses to species occurrence rates, with values closer to 1 indicating superior predictive performance (Wang et al., 2024). From the results of the single-model training groups, well-performing individual models were selected based on evaluation metrics. An Ensemble Model (EM) was generated using the weighted average method for subsequent prediction of suitable habitats for *C. arabica*.

### 2.4 Spatial pattern dynamics of suitable distribution areas for *C. arabica*

#### 2.4.1 Classification of suitable habitat grades

Data processing was performed using R. The raster projection results generated by the EM were imported into ArcGIS v10.8. Based on the raster suitability values calculated by EM, the raster cells were classified into suitable habitat grades, and the results were visualized. Specifically, the projected raster maps generated by EM were first imported into ArcGIS 10.8 for normalization. Using the reclassification function in the ArcToolbox, the raster suitability values (P) derived from the EM were categorized into four grades: unsuitable areas (0 ≤ P ≤ 0.3), low-suitable areas (0.3 < P ≤ 0.6), moderately suitable areas (0.6 < P ≤ 0.85), and highly suitable areas (0.85 < P ≤ 1). Classification based on grid suitability values effectively eliminates inconsistencies in classification criteria across different scenarios. This method was applied to the classification of suitable habitat rasters under both current and future climatic conditions.

#### 2.4.2 Changes in suitable habitats

Following the aforementioned method, presence/absence (0-1) matrices characterizing the potential geographic distribution of *C. arabica* under current and future climate scenarios were constructed to mitigate potential biases in probability values. In these matrices, suitable habitats (with values > 0.3) were coded as presence (1), while unsuitable areas (with values < 0.3) were coded as absence (0). Based on the transitions between 0 and 1 in the matrices, changes in suitable habitats were categorized into four types: 0→1 indicating newly gained suitable areas, 1→0 indicating lost suitable areas, 1→1 indicating retained suitable areas, and 0→0 indicating persistently unsuitable areas. Changes in the area corresponding to each category were then computed.

#### 2.4.3 Transitions of suitable habitats under current and future climate scenarios

The four types of suitable habitats of *C. arabica* under current and future climatic conditions were reclassified, and then raster calculations were performed as required by the analysis. The reclassification was assigned as follows, 1 for unsuitable areas, 2 for low-suitable areas, 3 for moderately suitable areas, 4 for highly suitable areas. The calculations were conducted using the following formula,


X=A×10+B

Note: A represents the raster code under the previous climatic conditions, and B represents the raster code under the subsequent climatic conditions.

### 2.5 Multivariate environmental similarity surface and most dissimilar variables analyses

Multivariate environmental similarity surface (MESS) analysis quantifies the environmental similarity between grid cells in the model training dataset and those in the novel prediction scenario. This approach not only delineates potential climatically dissimilar regions across the temporal and spatial scope of the prediction but also identifies key variables regulating species habitat suitability via limiting factor analysis. Specifically, limiting factors refer to the variables exerting the most substantial influence on model output variations, which are also defined as the most dissimilar variables (MOD). MESS calculation quantifies the degree of similarity between the set of predictive variables and the reference layer, when 0 < S < 100, a smaller value indicates a more significant difference between the climatic factors and the reference layer, S = 100 means there is no difference between the reference layer and the climatic factors, and S ≤ 0 indicates that at least one climatic factor value is outside the range of the reference layer during the specific period, with a larger negative value representing a higher degree of climatic anomaly (Zhang et al., 2023).

## 3 Results

### 3.1 Screening of environmental variables

A total of 29 environmental variables were selected for model construction in this study, encompassing 19 climatic, 7 soil factors and 3 topographic factors. Following screening, 11 environmental variables were retained for constructing the species distribution model, including 6 climatic factors (Bio1, Bio2, Bio3, Bio4, Bio11, Bio18), 3 topographic factors (elevation, slope, aspect), and 2 soil factors (Ref-bulk, soil pH) (**Table 1**).

**Table 1:** Description of environmental variables and their contribution ratio.

Environmental variable	Description	Code	Contribution ratio/%
Climate factors	Mean annual temperature (°C)	Bio1	10
	Mean diurnal range (°C)	Bio2	1.2
	Isothermality (bio3 = (bio1/bio7)×100)	Bio3	10.4
	Temperature seasonality (C of V)	Bio4	40.7
	Average temperature in coldest quarter (°C)	Bio11	18.5
	Precipitation in the warmest quarter (mm)	Bio18	3.4
Topographic factors	Elevation (m)	elev	1.7
	Slope (°)	Slope	0.8
	aspect	aspect	2.7
Soil factors	Top of bulk density	Ref_Bulk	0.5
	Top potential of hydrogen	T_pH-H_2_O	0.8

### 3.2 Model optimization

**Figure 2:**
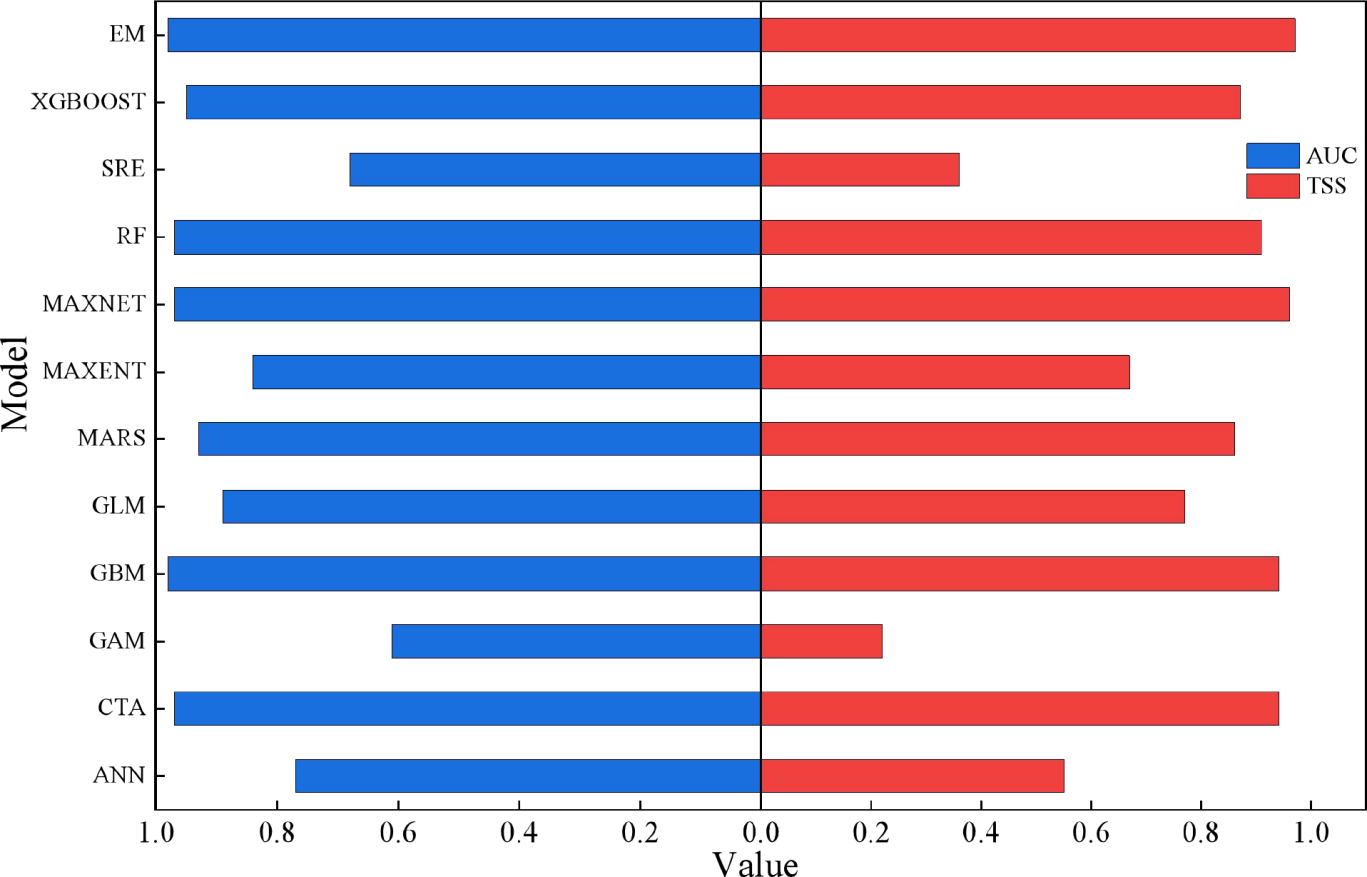
Comparison of TSS and AUC of solitary model and ensemble model.

The suitable habitats of all species were simulated using the Biomod2 package (4.2.5-2). Each of the 12 individual models was run 10 times repeatedly, and the evaluation results of TSS and AUC for each individual model were obtained, among which the FDA model failed to be constructed (**Figure 2**). Among all the candidate models, the ANN, GAM and SRE models performed most poorly in terms of AUC and TSS metrics, failing to pass the model accuracy validation. Conversely, the rest of the individual models all attained AUC values above 0.8 and TSS values beyond 0.6, demonstrating superior predictive performance. To further optimize the algorithm, models with AUC > 0.90 and TSS > 0.80 were selected, including CTA, GBM, MARS, MAXNET, RF and XGBOOST, for the construction of the EM. The ensemble model yielded a mean AUC of 0.98 and a mean TSS of 0.97, both of which were higher than those of the individual models. The model accuracy test results were excellent, suggesting that the ensemble model provides more accurate and reliable predictions of the potential geographical distribution of *C. arabica*.

### 3.3 Response curve analysis

**Figure 3:**
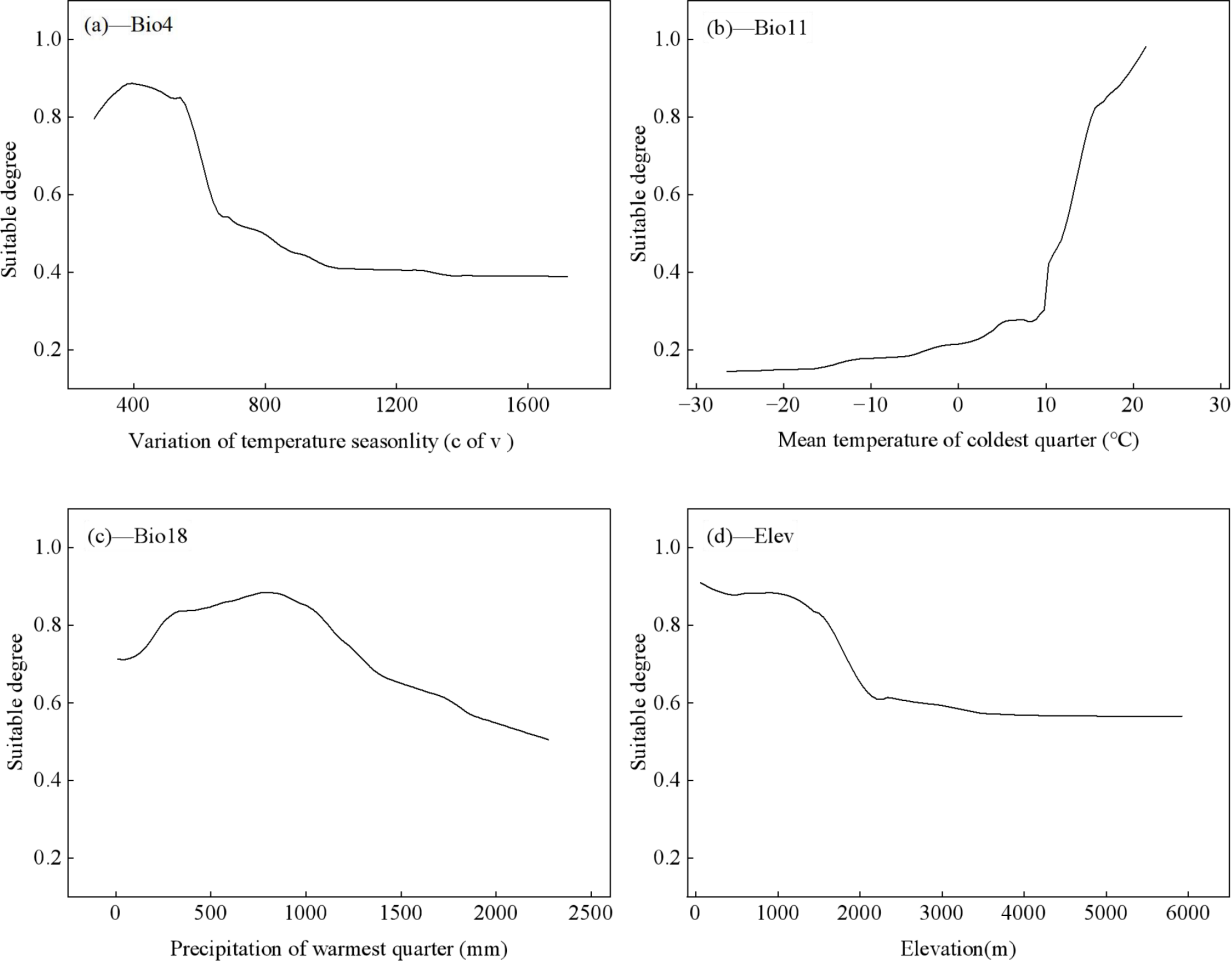
Response curves for important environmental variables. **(a)** Bio4; **(b)** Bio11; **(c)** Bio18; **(d)** Elevation.

Based on the ensemble model analysis of the selected key environmental factors, the temperature seasonality coefficient exerted the greatest influence on the distribution of *C. arabica*, followed by the mean temperature of the coldest quarter, precipitation of the warmest quarter, and elevation (**Figure 3**). The suitability of *C. arabica* exhibited a trend of first increasing and then decreasing as the temperature seasonality coefficient increased (**Figure 3a**). Suitability reached the optimal level when the temperature seasonality coefficient was 380, and began to decline when the coefficient exceeded 570. The average temperature of the coldest quarter spanned a range of -26°C to 21.45°C (**Figure 3b**). As temperature increased, survival suitability increased, with optimal suitability observed when the mean temperature of the coldest quarter was between 13°C–21°C. Survival suitability also increased with increasing precipitation in the warmest quarter. Suitability reached a maximum of 0.88 when precipitation ranged from 700mm to 850mm, beyond 850 mm, suitability began to decrease, dropping to 0.5 at 1, 790mm and ceasing to change thereafter (**Figure 3c**). Elevation was another key factor affecting survival suitability. As indicated by the response curve (**Figure 3d**), the probability of occurrence of *C. arabica* gradually decreased with increasing elevation, declining sharply when elevation reached 1800m. In summary, conditions conducive to the growth of *C. arabica* include a temperature seasonality coefficient of 380, a mean temperature of the coldest quarter between 13°C–21°C, precipitation of the warmest quarter between 700mm–850mm, and elevation below 1, 800m.

### 3.4 Prediction of current suitable habitats

**Figure 4:**
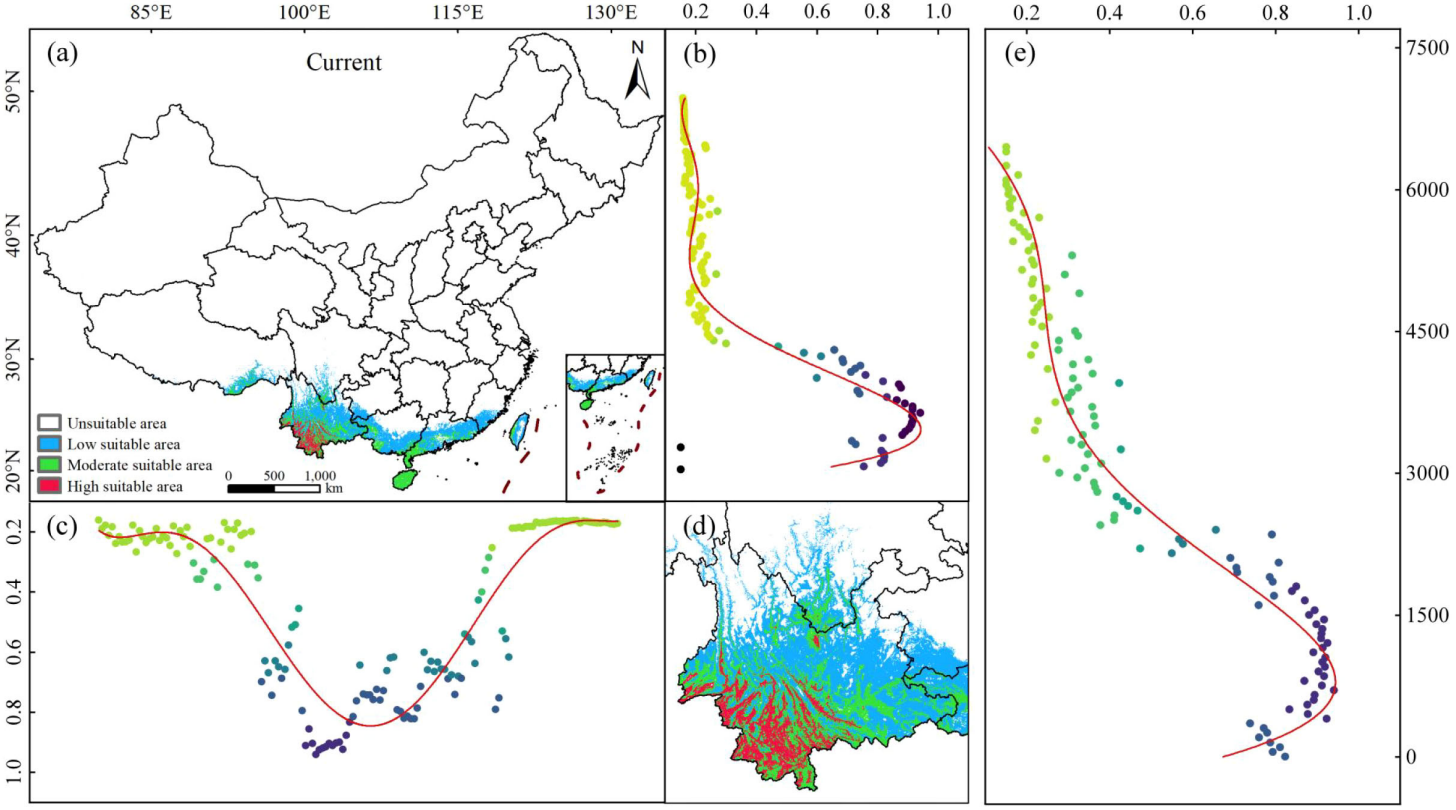
Distribution of current suitable habitats of *C. arabica.* **(a)** Distribution of current suitable habitats; **(b)** Latitude of current suitable habitats; **(c)** Longitude of current suitable habitats; **(d)** Current suitable area of Yunnan province; **(e)** Elevation of current suitable habitats.

Currently, *C. arabica* is primarily distributed in southwestern and southeastern China (**Figure 4a**), with a total suitable habitat area of 63.89 × 10^4^ km^2^, accounting for 6.65% of China’s total land area. Specifically, the area of low-suitable habitats is 38.98 × 10^4^ km^2^, representing 61.01% of the total suitable habitat area; moderately suitable habitats cover 23.61 × 10^4^ km^2^, making up 36.95%, highly suitable habitats occupy 1.3 × 10^4^ km^2^, accounting for 2.03% (**Table 2**). Low-suitable habitats are mainly distributed in southeastern Tibet, central Yunnan, southern Sichuan, southeastern Guangxi, central Guangdong, southeastern Fujian, and eastern Taiwan (26°N–30°N, 84°E–120°E). Moderately suitable habitats are concentrated across the entire Hainan, as well as in southwestern and southeastern Yunnan, southern Sichuan, parts of southeastern Tibet, parts of western Guangxi, parts of southern and southeastern Guangdong, and western Taiwan (18°N–29°N, 92°E–121°E). Highly suitable habitats are predominantly located in Xishuangbanna Prefecture, Pu’er City, Lincang City, Dehong Prefecture, Baoshan City, Honghe Prefecture, Yuxi City, and Chuxiong Prefecture in Yunnan Province (21°N–26°N, 98°E–102°E), with dense distributions in Xishuangbanna Prefecture, Pu’er City, and Lincang City (**Figures 4a–d**). The current suitable habitats of *C. arabica* exhibit a trend of decreasing suitability with increasing elevation. Moderately and highly suitable areas are mainly concentrated below 2, 400 m, with optimal suitability observed at elevations below 1, 800 m (**Figure 4e**).

**Table 2:** Area changes of *C. arabica* suitable habitats under future climate scenarios.

Climate scenario	Period	Area of unsuitable/ (× 10^4^ km^2^)	Area of low suitable/ (× 10^4^ km^2^)	Area of moderate suitable/ (× 10^4^ km^2^)	Area of high suitable/ (× 10^4^ km^2^)	Total suitable area/ (× 10^4^ km^2^)
Current	Current	897.27	39.98	23.61	1.3	63.89
SSP126	2030s	868.97	63.75	26.81	2.26	92.82
	2050s	859.09	68.87	31.43	2.39	102.69
	2070s	883.33	58.13	18.24	2.09	78.46
	2090s	849.81	81.23	28.67	2.08	111.98
SSP245	2030s	866.73	66.44	26.26	2.35	95.05
	2050s	834.59	94.68	30.09	2.42	127.19
	2070s	849.50	80.91	29.89	1.47	112.27
	2090s	807.74	117.38	33.86	2.80	154.04
SSP370	2030s	883.21	56.02	19.18	3.38	78.58
	2050s	866.16	67.94	24.99	2.69	95.62
	2070s	830.74	100.79	28.51	1.74	131.04
	2090s	782.56	151.12	26.40	1.70	179.22
SSP585	2030s	867.97	67.15	24.05	2.61	93.81
	2050s	829.16	98.12	32.04	2.47	132.63
	2070s	818.82	111.19	30.58	1.2	142.97
	2090s	777.50	151.80	31.15	1.34	184.29

### 3.5 Prediction of future suitable habitats

**Figure 5:**
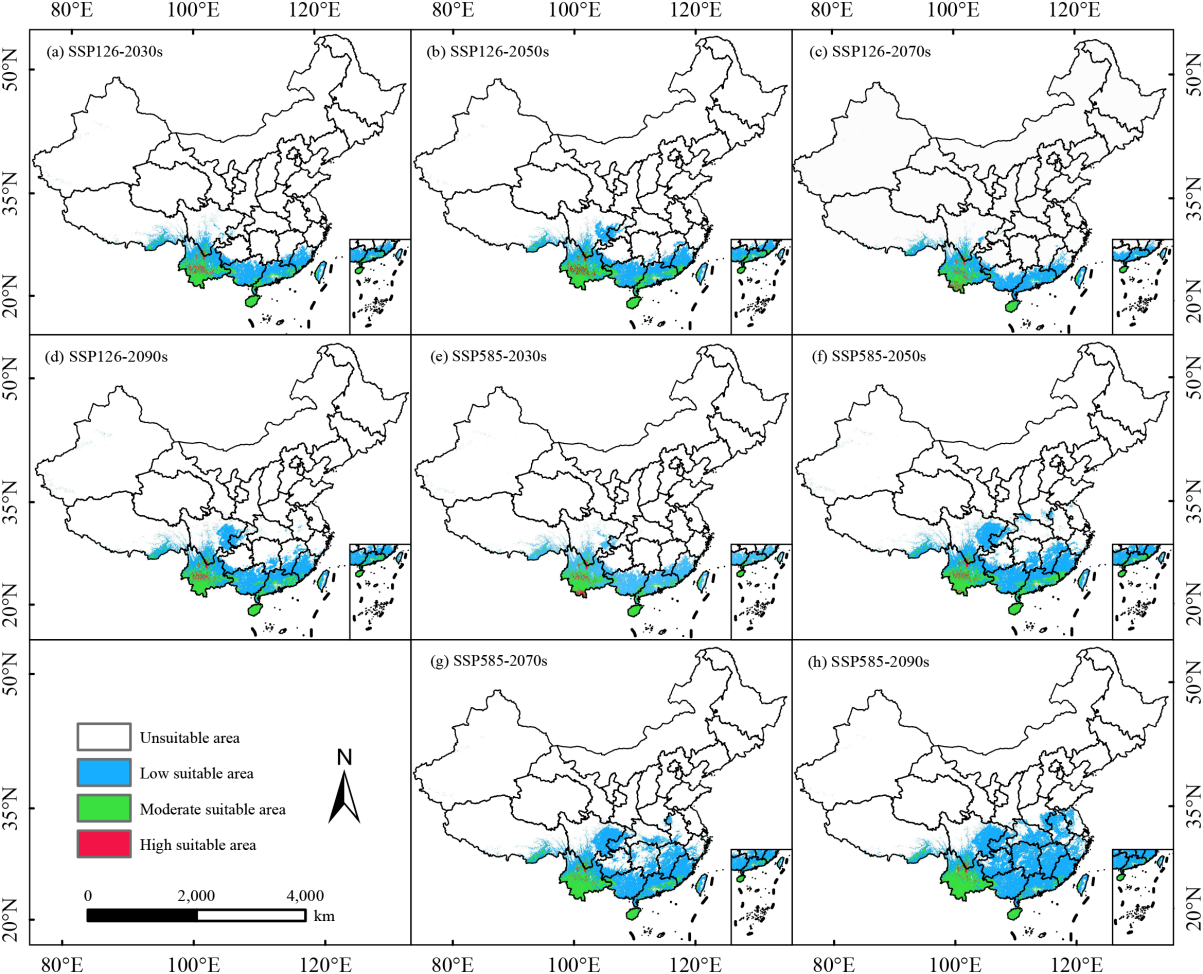
Predicted potential suitable habitats for *C. arabica* in China under different climate scenarios **(a–h)**.

Under future climate scenarios, the area of unsuitable habitats for *C. arabica* is continuously shrinking, while the total suitable habitat area shows an increasing trend compared to the current period (**Figure 5**). However, the trends vary among different suitability grades (**Table 2**). Over time, the total suitable habitat area is projected to expand, particularly under SSP585, where it exhibits higher suitability, with the total suitable habitat area reaching a maximum of 184.29 × 10^4^ km^2^ in the 2090s (**Figure 5h**). In contrast, the total suitable habitat area under SSP126 scenario shows a small increase, rising from 92.82 × 10^4^ km^2^ in the 2030s to 111.98 × 10^4^ km^2^ in the 2090s (**Figures 5a–d**). Under the SSP245 and SSP370 scenarios, the total suitable habitat area of *C. arabica* will increase over time, while the highly suitable areas will show a decreasing trend (**Supplementary Figure 1**). The area of low-suitable habitats will reach a maximum of 151.80×10^4^km^2^ under SSP585 in the 2090s (**Figure 5h**). Except for SSP585 in the 2070s (1.2 × 10^4^ km^2^), the area of highly suitable habitats will show a fluctuating increasing trend in other climate scenario periods. Moderately suitable habitats will also exhibit a fluctuating increasing trend (**Table 2**). Unsuitable areas are widely distributed in high-latitude regions north of the current suitable habitats. Under all future climate scenarios, their area will remain stable at 841.69 × 10^4^ km^2^, accounting for more than half of the total suitable habitat area. Overall, the results indicate that under future climatic conditions, the area of highly suitable habitats for *C. arabica* will show a fluctuating trend of increase-decrease-increase, while the overall suitable distribution area is projected to expand, suggesting that *C. arabica* will maintain good suitability under future climate conditions.

### 3.6 Transitions of suitable habitats

**Figure 6:**
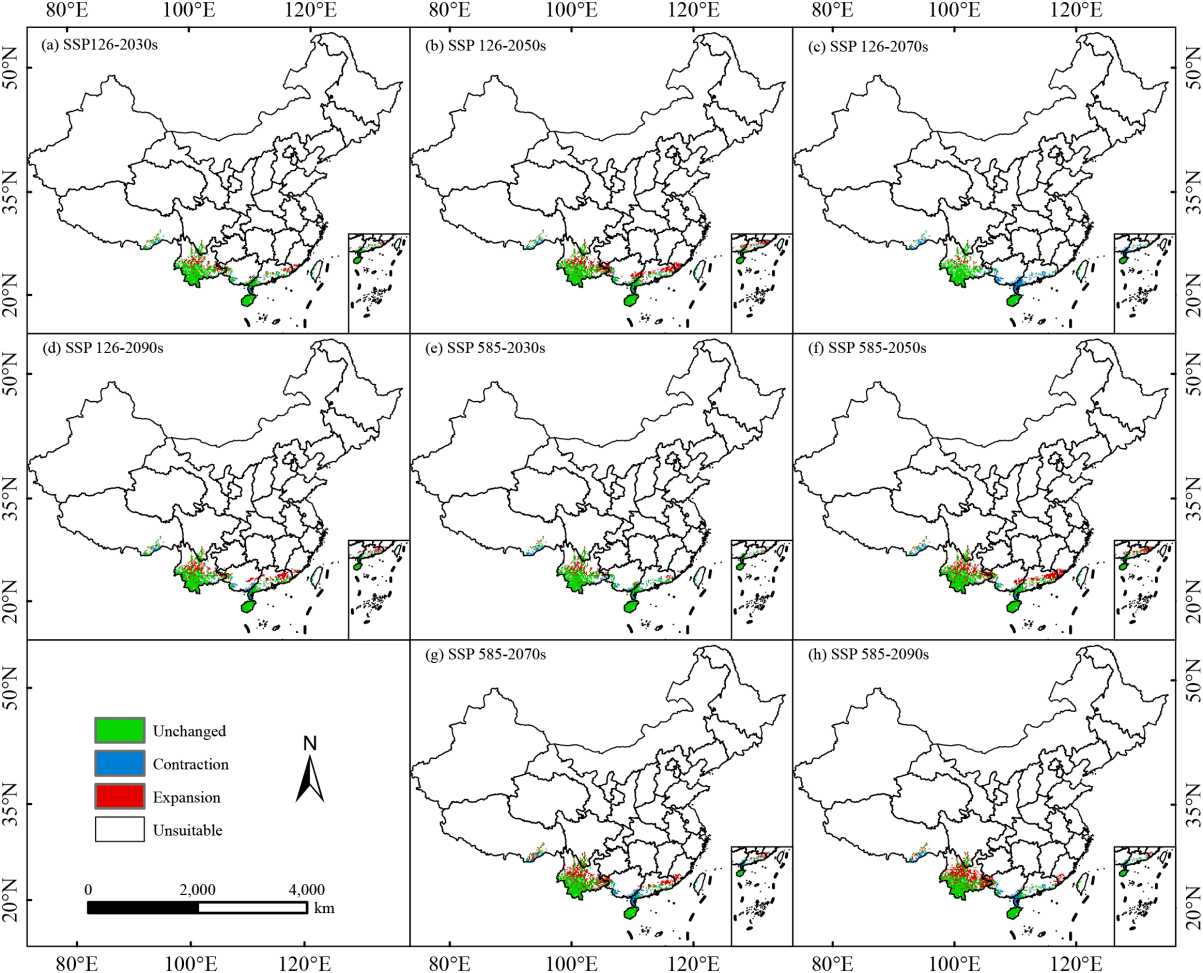
Spatial variations in the moderate and high suitable for *C. arabica* in China under different climate scenarios **(a–h)**.

Significant differences exist in the spatiotemporal dynamics of moderately and highly suitable habitats of *C. arabica* under different climate scenarios. In the SSP245 scenario, the suitable habitat area showed the largest expansion in the 2090s, reaching 13.64 × 10^4^ km^2^ (**Supplementary Figure 2d**), under the SSP126 scenario, the change in suitable habitat area was relatively stable, with the most significant increase occurring in the 2050s, 43.97% expansion rate, corresponding to a newly added area of 10.75 × 10^4^ km^2^ (**Figure 6b**). In the 2070s, the largest area loss (7.31 × 10^4^ km^2^) and the smallest newly added area (2.25 × 10^4^ km^2^) were observed, while the area loss rates in the 2030s (**Figure 6a**) and 2090s (**Figure 6d**) were lower than that in the 2070s (**Figure 6c**). Under the SSP585 scenario, the expansion rate of suitable habitats gradually increased over time, while the loss rate first decreased and then increased. The expansion peaked in the 2090s, with an expansion rate of 49.04% (11.99 × 10^4^ km^2^), and the loss rate in this period (20.33%) was significantly higher than in other periods, corresponding to a lost area of 4.97 × 10^4^ km^2^ (**Table 3**). Under the SSP126 and SSP585 scenarios, the expanded areas were concentrated in central Yunnan and its border with Guangxi, as well as the border area between Guangdong and Fujian; the lost areas were mainly in Guangxi, southern Guangdong, and southeastern Tibet, the stable areas were concentrated in southwestern Yunnan and Hainan (**Figure 6**). Under the SSP245 and SSP370 scenarios, the stable areas (southwestern Yunnan and Hainan) increased over time; the contracted areas (Nyingchi in Tibet and the border area between Guangxi and Guangdong) gradually decreased; the expanded areas (central Yunnan and the border area between Guangdong and Fujian) showed fluctuating changes (**Supplementary Figure 2**).

**Table 3:** Area changes of *C. arabica* moderate and high suitable habitats under future climate scenarios.

Climate scenario	Period	Total suitable area/ (× 10^4^ km^2^)	Retained area/ (× 10^4^ km^2^)	Contraction area/ (× 10^4^ km^2^)	Contraction rate/%	Expansion area/ (× 10^4^ km^2^)	Expansion rate/%
Current	Current	24.45	–	–	–	–	–
SSP126	2030s	28.49	22.28	2.17	8.88	6.21	25.40
	2050s	33.12	22.37	2.08	8.51	10.75	43.97
	2070s	19.39	17.14	7.31	29.90	2.25	9.20
	2090s	30.09	21.36	3.09	12.64	8.73	35.71
SSP245	2030s	27.8	20.37	4.07	16.65	7.43	30.39
	2050s	31.47	21.53	2.92	11.94	9.94	40.65
	2070s	30.63	22.08	2.37	9.69	8.55	34.97
	2090s	35.72	22.08	2.37	9.69	13.64	55.79
SSP370	2030s	21.65	18.30	6.16	25.19	3.35	13.70
	2050s	26.94	19.54	4.91	20.08	7.40	30.27
	2070s	29.28	20.32	4.13	16.89	8.96	36.65
	2090s	27.46	20.09	4.36	17.83	7.37	30.14
SSP585	2030s	26.15	21.18	3.27	13.37	4.97	20.33
	2050s	33.79	21.91	2.54	10.39	11.88	48.59
	2070s	30.72	19.54	4.54	18.57	11.18	45.73
	2090s	31.47	19.48	4.97	20.33	11.99	49.04

**Figure 7:**
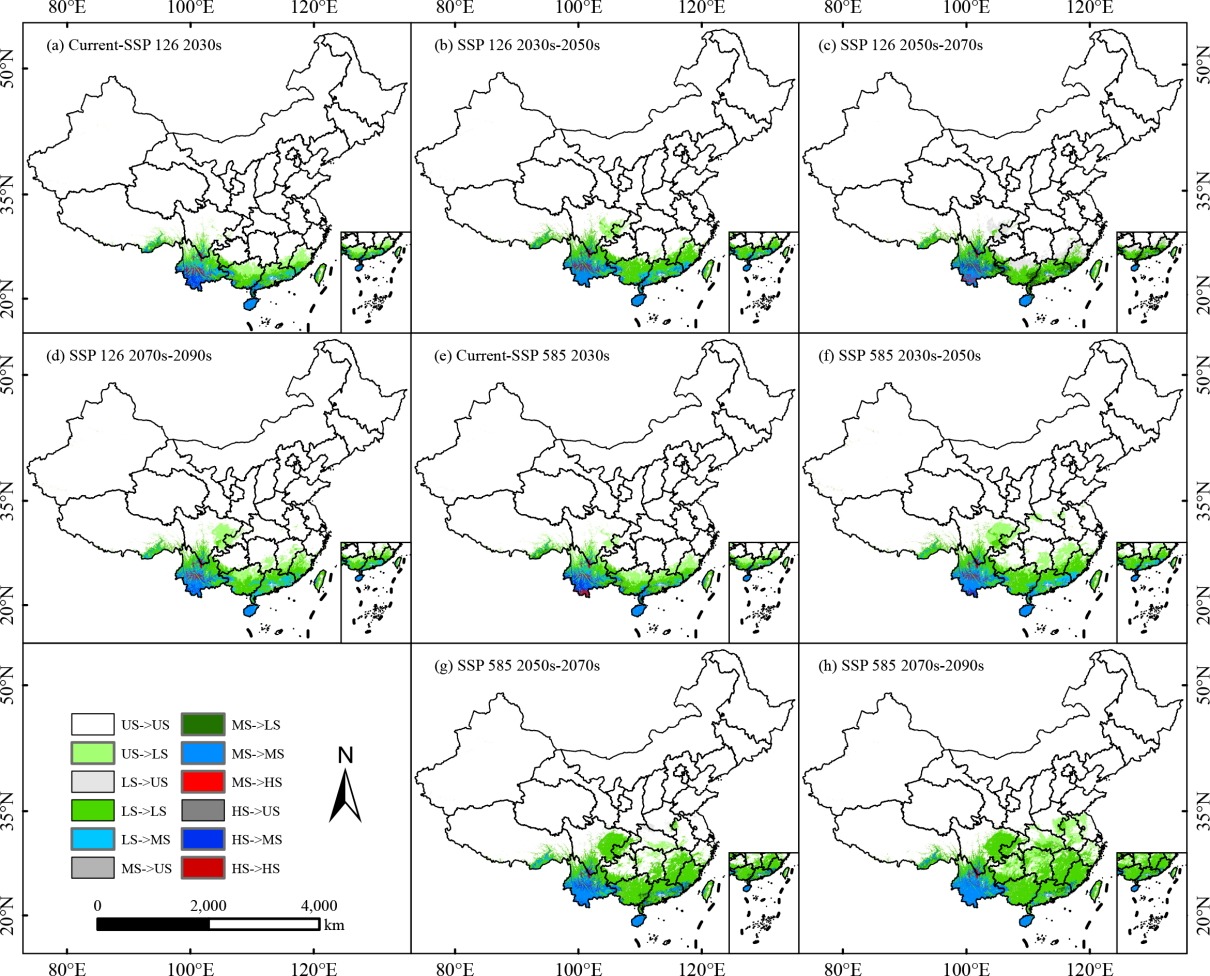
Different suitable habitats transfer of *C. arabica* in China under future climate scenarios **(a–h)**. The US, LS, MS, and HS represent unsuitable, low suitable, moderate suitable and high suitable.

**Figure 8:**
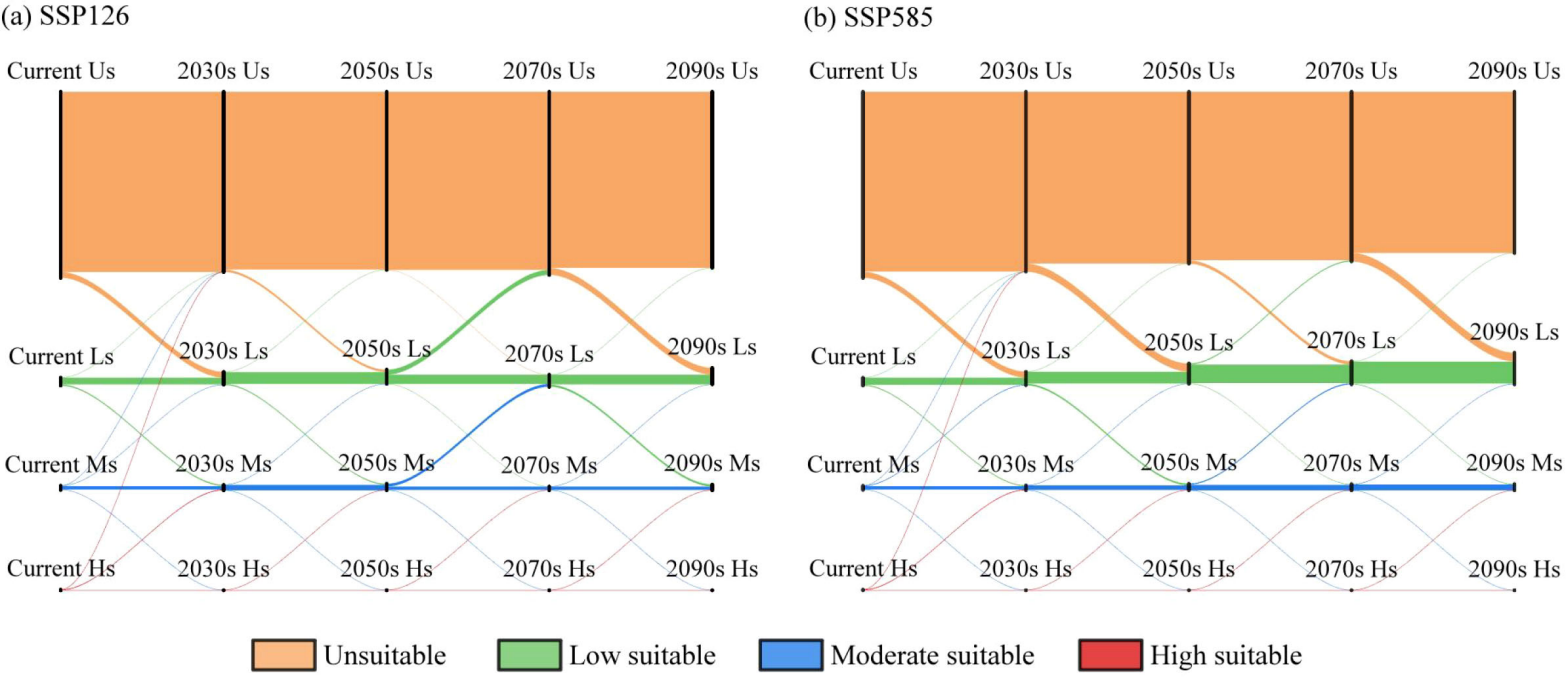
Area transfer of *C. arabica* in China under future climate scenarios. **(a)** Area transition under the SSP126 scenario; **(b)** Area transition under the SSP585 scenario.

From the current period to future scenarios under different climate conditions, changes in the suitable areas of *C. arabica* across different decades primarily occur at the boundaries between different suitability grades. Among all grades, the proportion of unchanged area is the highest, with the largest unchanged area found in unsuitable regions, though this area shows a gradual decreasing trend with climate change (**Figures 7**, **8**). Under the SSP126 scenario, the unchanged area of low and moderately suitable regions fluctuates and increases, while that of highly suitable regions gradually decreases. During the current period to the 2030s, apart from unchanged areas, the most prominent change is the conversion from unsuitable areas to low-suitable areas (28.47 × 10^4^ km^2^), involving the border region between Yunnan and Guizhou, northern Guangxi, northern Guangdong, southern Jiangxi, and central Fujian (**Figure 7a**). From the 2030s to the 2050s and 2070s to the 2090s, the extent of changes in suitable habitats was relatively small. The most prominent shift remained the conversion from unsuitable areas to low-suitable areas, with the area changes being 11.69 × 10^4^ km^2^ and 10.43 × 10^4^ km^2^, respectively, primarily in eastern Sichuan and western Chongqing (**Figures 7b, d**). During the 2050s to the 2070s, the most notable change is the conversion from low-suitable areas to unsuitable areas (24.41 × 10^4^ km^2^), affecting eastern Sichuan, northern Chongqing, northern Guangxi, southeastern Jiangxi, and central Fujian. Additionally, the area of conversion from moderately suitable areas to low-suitable areas is also significant (13.54 × 10^4^ km^2^), including parts of Yunnan, Guangxi, Guangdong, and Fujian (**Figure 7c**).

Under the SSP585 scenario, the unchanged area of highly suitable habitats gradually decreases over time, while the areas of low and moderately suitable habitats gradually increase. From the current period to the 2030s, southwestern Yunnan will transitions to moderately suitable habitats (**Figure 7e**). From the 2030s to the 2090s, the most prominent change is the conversion from unsuitable areas to low-suitable areas, with regions south of the Yangtze River will becoming low-suitable habitats (**Figures 7f–h**).

Under the SSP245 and SSP370 scenarios, the unchanged area of unsuitable habitats accounts for the largest proportion; however, this unchanged area gradually decreases over time, while the unchanged areas of low and moderately suitable habitats show a gradual increasing trend, with the opposite pattern observed for the unchanged area of highly suitable habitats (**Supplementary Figures 3**, **4**). The most prominent change is the conversion from unsuitable areas to low-suitable areas, with the largest conversion area (49.47 × 10^4^ km^2^) occurring during the 2070s–2090s under SSP370, where regions such as Hunan, Hubei, Zhejiang, Jiangsu, and Anhui will transition to low-suitable habitats (**Supplementary Figure 3h**). Under the SSP245 scenario, from the current period to the 2030s, parts of southwestern Yunnan will shift from moderately suitable habitats to highly suitable habitats (**Supplementary Figure 3a**). From the 2050s to the 2070s, parts of northeastern Sichuan and central Hunan exhibit a distinct trend of conversion from low-suitable areas to unsuitable areas (**Supplementary Figure 3c**).

Overall, the results demonstrate that under future climatic conditions, as temperatures rise, *C. arabica* will expand toward higher-latitude regions north of its current distribution, with unsuitable areas gradually transitioning to low-suitable areas.

### 3.7 MESS and MoD analysis

**Figure 9:**
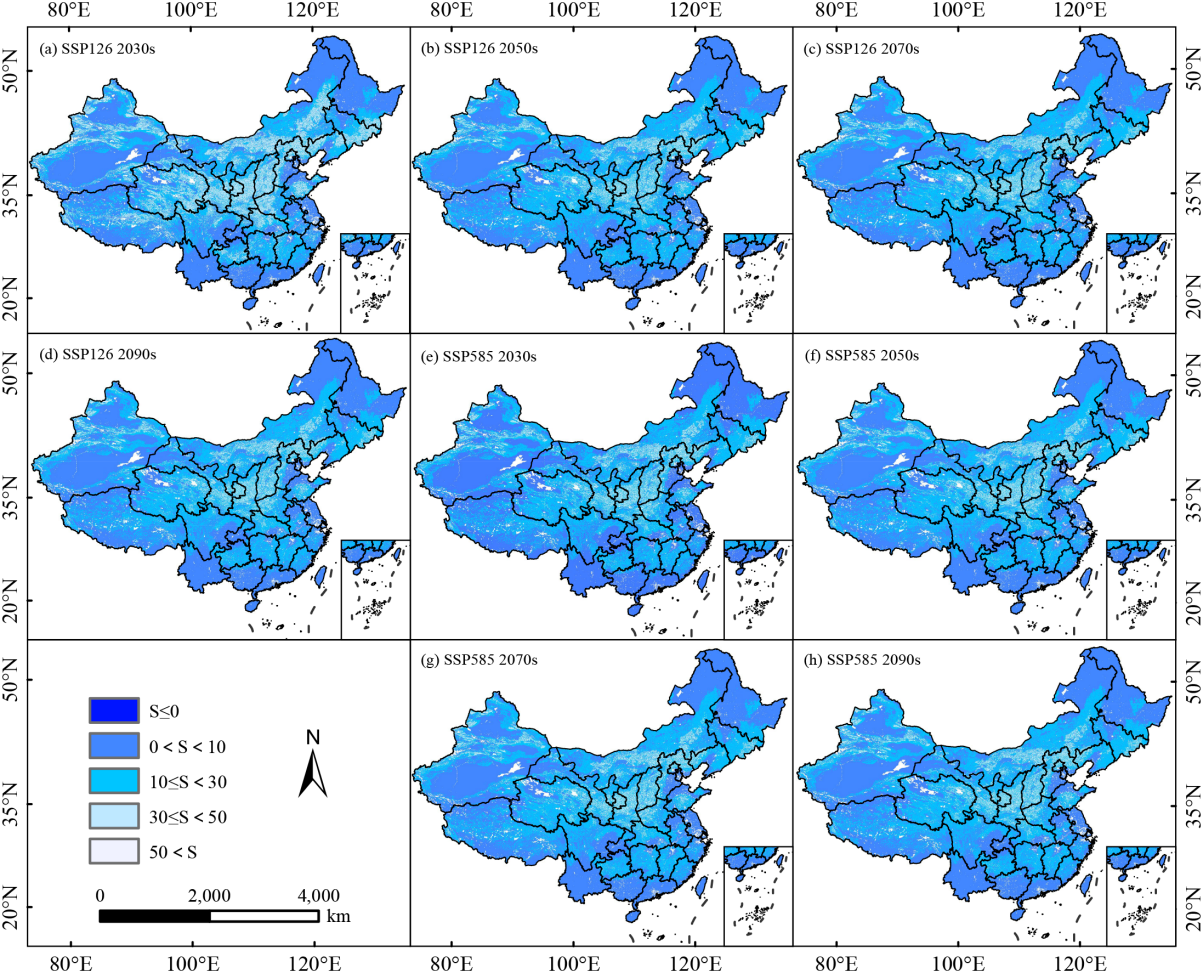
Multivariate environmental similarity surface variable analysis for *C. arabica* in China under different combinations of climate change scenarios **(a–h)**.

**Figure 10:**
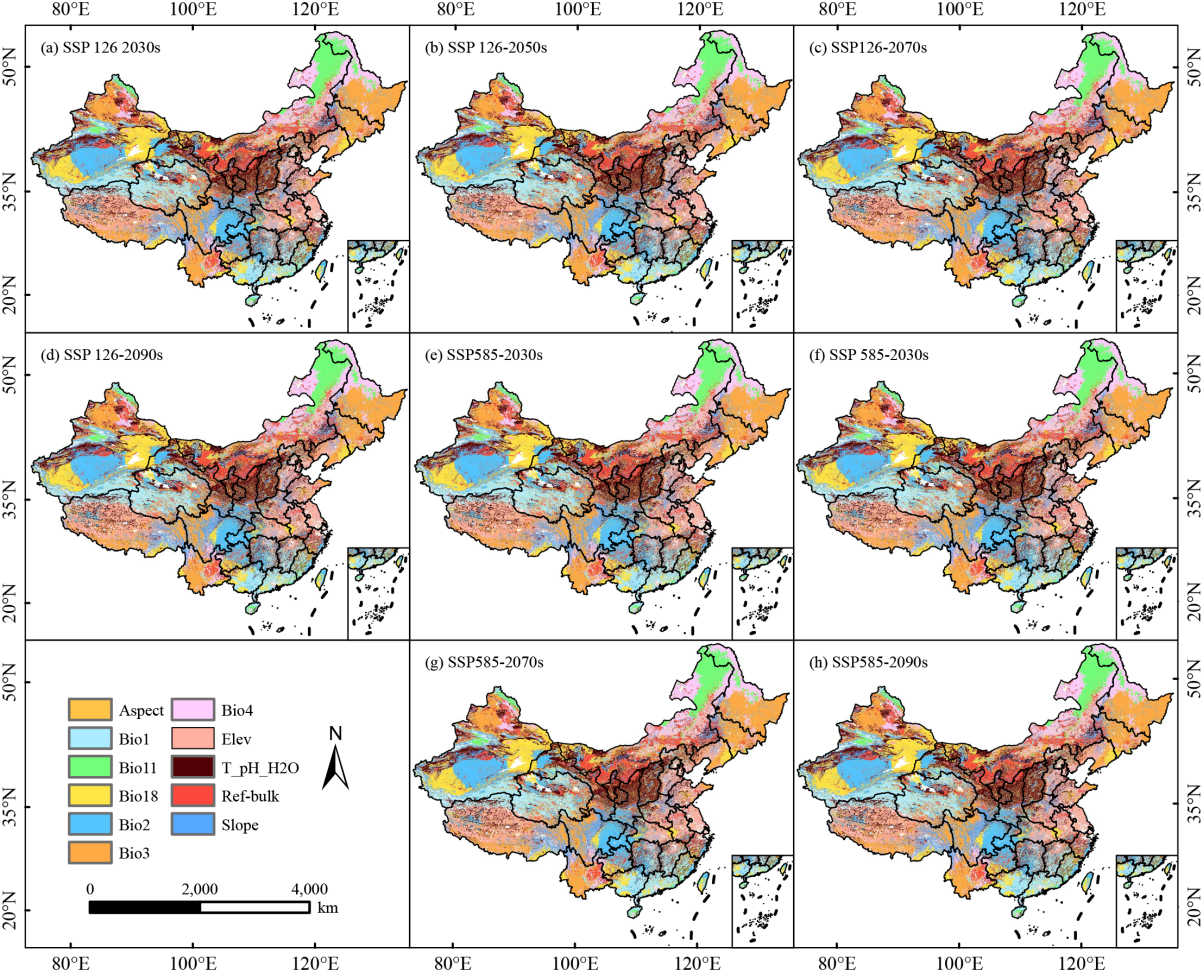
Most dissimilar variable analysis for *C. arabica* in China under different combinations of climate change scenarios **(a–h)**.

Results from the MESS analysis indicated that, under the potential climate scenarios across four future periods, the degree of climatic anomaly exhibits a decreasing trend from west to east across the study area, regions with extreme climatic anomalies nationwide (S<0) were negligible (**Figure 9**; **Supplementary Figure 5**). The range of suitable habitats for *C. arabica* in China was largely confined to regions characterized by climatic anomalies (0<S<10). Compared with the current period, among all scenarios within this region, isothermality (Bio3) was the most divergent environmental variable with the highest contribution rate, followed by annual mean temperature (Bio1), mean temperature of the coldest quarter (Bio11), precipitation of the warmest quarter (Bio18), temperature seasonality coefficient (Bio4), and soil bulk density (Ref-bulk), which exhibited a scattered distribution pattern in Yunnan Province (**Figure 10**; **Supplementary Figure 6**).

## 4 Discussion

Currently, most studies employ a single model for species distribution prediction and analysis. Although single models often yield high evaluation scores, such elevated values do not necessarily correspond to superior predictive performance; instead, model selection and interpretation should be tailored to the biological and ecological characteristics of the target species. The uncertainty inherent in single-model approaches is unavoidable, which can lead to substantial discrepancies in species distribution simulations. In some cases, these discrepancies may even exceed the variations in projection outcomes driven by differences in emission scenarios and combinations of climatic factors (Hao et al., 2019). One of the advantages of ensemble models lies in avoiding the selection of a single model, thereby eliminating or minimizing biases associated with model selection. By integrating results from multiple individual models, ensemble approaches allow each constituent model to exert its respective strengths, which effectively improves predictive reliability and overall model accuracy. Therefore, ensemble modeling represents a more robust and preferable framework, as it can reduce uncertainty derived from individual model structures to a certain extent and yields more reliable projections in species distribution modeling (Pais et al., 2020). To reduce errors, the Biomod2 package in R software was used to evaluate the models through TSS and AUC values, thereby minimizing the inherent errors in the models, and further enabling more accurate simulation of geographical distributions and inference of the key environmental factors influencing species distribution (Zhang et al., 2024). The results showed that both TSS and AUC values of the ensemble model were significantly higher than those of the single models, effectively improving the accuracy of model predictions. This indicates that ensemble model integration has important application value in research on biological distribution prediction. Nevertheless, several limitations and sources of uncertainty exist in the model used in this study. First, there are limitations in the occurrence records of *C. arabica*. Constrained by field survey conditions, although the collected records cover the main planting areas of *C. arabica*, the insufficient samples in some marginally suitable habitats may result in slight deviations in the model’s prediction accuracy for those regions. Second, uncertainty exists in the climate data. Despite strict validation, WorldClim data still contain certain spatial interpolation errors, which may have minor impacts on the correlation analysis between the suitable habitats of *C. arabica* and climatic factors. Third, the model is restricted by its inherent assumption. Species distribution models are based on the niche conservatism hypothesis, and do not fully consider anthropogenic factors such as artificial cultivation and variety improvement that affect the distribution of *C. arabica*. Future studies can further incorporate anthropogenic disturbance factors to optimize the model.

Climatic factors are key determinants of plant geographical distribution (Chen et al., 2023). Climate change-induced alterations in temperature and precipitation patterns have profoundly impacted multiple aspects of biodiversity (Bellard et al., 2012). Our results indicated that the suitability of *C. arabica* gradually decreases when the temperature seasonality coefficient exceeds 380 and the mean temperature of the coldest quarter drops below 13°C. This aligns with the well-established fact that temperature is a critical limiting variable for coffee distribution suitability (DaMatta and Ramalho, 2006). Precipitation is another important factor influencing *C. arabica*. Excessive rainfall during its flowering period can cause flower abscission and hinder fruiting, while insufficient precipitation may lead to drought, thereby reducing fruiting rates (Tavares et al., 2018; Tun et al., 2021). The suitability of *C. arabica* reaches an optimal level of 0.88 when precipitation in the warmest quarter ranges from 700mm to 850mm, and begins to decline when precipitation exceeds 850 mm. Notably, the peak flowering period of *C. arabica* (May-June annually) coincides with the hottest months of the year, such that either excessive or insufficient precipitation during this period is unfavorable for its growth. Furthermore, the suitability of *C. arabica* remains above 0.8 at elevations between 0m and 1800m, starts to decrease above 1800m, and drops below 0.5 at elevations exceeding 2400m, which is detrimental to its growth. This may be attributed to large diurnal temperature variations in high-altitude regions, where both temperature and precipitation are likely lower compared to low-altitude areas, thereby creating unfavorable conditions for *C. arabica* (Li et al., 2020). Similar studies by other scholars have concluded that the optimum elevation range for *C. arabica* in Yunnan is 800–1500 m, which is consistent with the findings of the present study (Zhang et al., 2021). Climate warming will alter the suitable habitats of *C. arabica.* Temperature, precipitation and elevation are the dominant factors, determine the potential geographical distribution range of *C. arabica* on a large scale, yet the constraints posed by other topographic and soil variables should not be underestimated.

**Figure 11:**
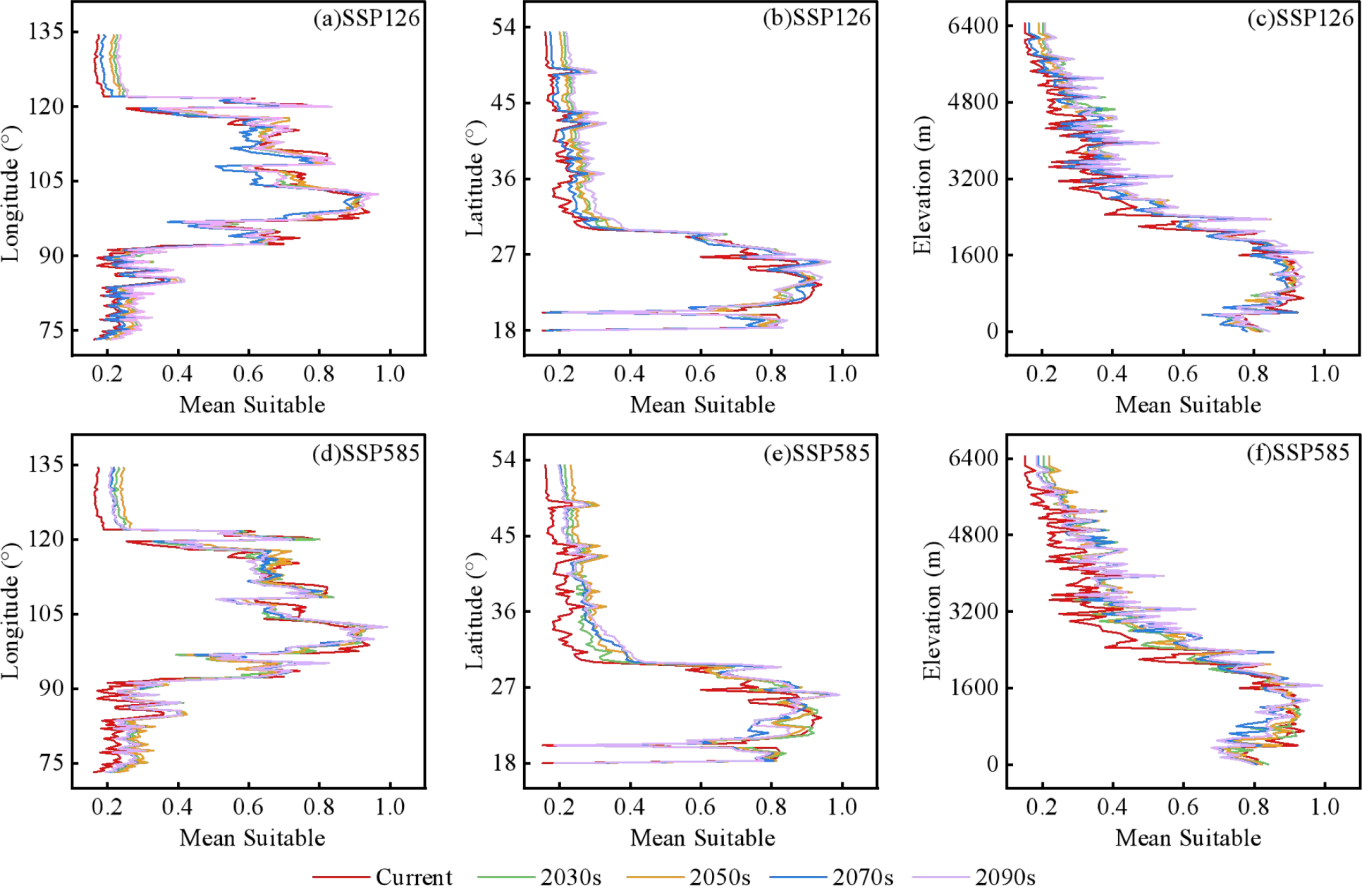
Variation of latitude, longitude and elevation of *C. arabica* in China under different combinations of climate change scenarios **(a–f)**.

Under current climatic conditions, *C. arabica* occurs mainly in southern and southwestern China under present climatic scenarios, while its highly suitable habitats are centered in southwestern Yunnan Province (**Figure 4**). Under future climate scenarios, the overall changing trend of *C. arabica* suitable habitats is consistent (**Figure 5**), may expand toward regions with higher latitudes (**Figure 11**; **Supplementary Figure 7**). This finding is consistent with the conclusions of other studies, which suggest that climate warming may drives species to migrate and expand toward higher latitudes (Chen et al., 2011). For future climate scenarios, moderately and highly suitable habitats of *C. arabica* is projected to the north of their current distribution, while the areas where habitats will vanish will be concentrated in the east of the present suitable range, with the expansion rate of suitable habitats exceeding that of contraction (Jinga and Palagi, 2020). Under the SSP585-2090s scenario, the total area of suitable habitats for *C. arabica* increases significantly, but the contracted area also increases simultaneously. This finding further confirms that climate change exerts a strong impact on the distribution of *C. arabica*, which is comparatively sensitive to climatic variations (Fenollosa and Munné-Bosch, 2018; Gebrewahid et al., 2020). Over the same period, the spatial variation range of C. arabica suitable habitats expands with the escalation of RCP concentrations, and the impacts of climate change become increasingly pronounced. Under the same RCP, the changes in *C. arabica* suitable habitats are positively correlated with time. Currently, multiple studies have predicted that the suitable cultivation area of coffee will increase in regions such as South America and Asia, with a shift toward higher elevations, while high-latitude regions may be less affected by rising temperatures and changes in rainfall patterns (Davis et al., 2012; Bunn et al., 2015), which is consistent with the findings of this study.

The MESS and MOD analysis serves as a robust methodological approach for quantifying the magnitude of climatic discrepancies between future and current conditions. Among these two analytical tools, MOD is capable of identifying the climatic variables with the most pronounced anomalies, which are typically the key drivers underpinning shifts in species distribution. Analyses of the MESS and MOD revealed that isothermality (Bio3) was the dominant climatic factor driving anomalies affecting the growth and development of *C. arabica*, followed by annual mean temperature (Bio1) and mean temperature of the coldest quarter (Bio11). These findings were consistent with those of previous studies. A growing body of research has demonstrated that climate change will drive the shift of *C. arabica* suitable habitats toward regions of higher elevations and latitudes, with its geographical distribution primarily constrained by temperature, precipitation factors (Zhang et al., 2021; Zhu et al., 2021; Li et al., 2024). In addition, *C. arabica* is a perennial species that thrives in cool, humid, and wind-sheltered regions, and its geographical distribution is more notably constrained by extreme temperatures.

## 5 Conclusions

Global climate change is an indisputable fact, which has significantly affected plant distribution patterns. To investigate the impacts and changing trends of global warming on the potential suitable habitats of *C. arabica* in China, as well as to identify the key influencing factors, we employed the Biomod2 ensemble modeling approach. Through model optimization, we simulated the potential suitable habitats of *C. arabica* under current climatic conditions, predicted the distribution and changing trends of suitable habitats across different periods under future climatic conditions, and analyzed the degree of climatic anomalies in the distribution areas using MESS and MoD methods. The results showed that under current climatic conditions, the suitable habitats of *C. arabica* are mainly distributed in Yunnan, Hainan, Guangxi, and Guangdong, with southwestern Yunnan being the highly suitable area. Under future climatic conditions, large areas of suitable habitats will emerge in regions south of the Yangtze River; particularly under the SSP585-2090s, the area of suitable habitats is projected to increase by 22.79% to 188.44% compared to the current period. Across different future periods, the moderately and highly suitable areas of *C. arabica* are regions with significant climatic anomalies, and the most dissimilar factors include Bio3, Bio1, Bio18, Bio4, Bio11 and soil bulk density; newly emerging suitable areas exhibit a relatively low degree of climatic anomaly. In addition, the geographical boundaries of different suitability grades show significant changes, with the area of low-suitable habitats showing the most prominent changes. In terms of the overall pattern of total suitable habitats, *C. arabica* will maintain good suitability in China in the future, with highly suitable areas remaining concentrated in Yunnan. Accurate simulation of its distribution dynamics and future patterns provides a scientific basis for climate change adaptation strategies and efficient utilization of germplasm resources of *C. arabica.*

Species distribution predictions based on models need to account for the influences of different models on species distributions, as well as uncertainties arising from these models. Additionally, the relationship between species’ intrinsic traits and abiotic factors can affect prediction outcomes. Variations in spatial resolution may also lead to biases in the prediction of environmental variables. Furthermore, factors including land use patterns and human activities are likely to exert an influence on the distribution of species suitable habitats. Therefore, future studies should incorporate more influencing factors and more specific information on species growth and development to ensure that prediction results are more accurate and reliable.

## Data Availability

The original contributions presented in the study are included in the article/**Supplementary Material**. Further inquiries can be directed to the corresponding authors.

## Supplementary material

Supplementary Figure S1 Predicted potential suitable habitats for *C. arabica* in China under different climate scenarios **(a–h)**.

Supplementary Figure 2 Spatial variations in the moderate and high suitable for *C. arabica* in China under different climate scenarios **(a–h)**.

Supplementary Figure 3 Different suitable habitats transfer of *C. arabica* in China under future climate scenarios **(a–h)**.

Supplementary Figure 4 Area transfer of *C. arabica* in China under future climate scenarios. **(a)** Area transition under the SSP245 scenario; **(b)** Area transition under the SSP370 scenario.

Supplementary Figure 5 Multivariate environmental similarity surface variable analysis for *C. arabica* in China under different combinations of climate change scenarios **(a–h)**.

Supplementary Figure 6 Most dissimilar variable analysis for *C. arabica* in China under different combinations of climate change scenarios **(a–h)**.

Supplementary Figure 7 Variation of latitude, longitude and elevation of *C. arabica* in China under different combinations of climate change scenarios **(a–f)**.
