# Supplementary material for: Climate change provides opportunities for the cultivation of Coffea arabica in China, an integrated model analysis based on biomod2
Source: Front Plant Sci. 2026 Mar 20;17:1806108. doi: 10.3389/fpls.2026.1806108 (PMC13047709; doi:10.3389/fpls.2026.1806108)
Supplement: Supplementary Figure S1 — Predicted potential suitable habitats for C. arabica in China under different climate scenarios (a–h). [file DataSheet1.zip › Supplementary file/Captions for all supplementary.docx]

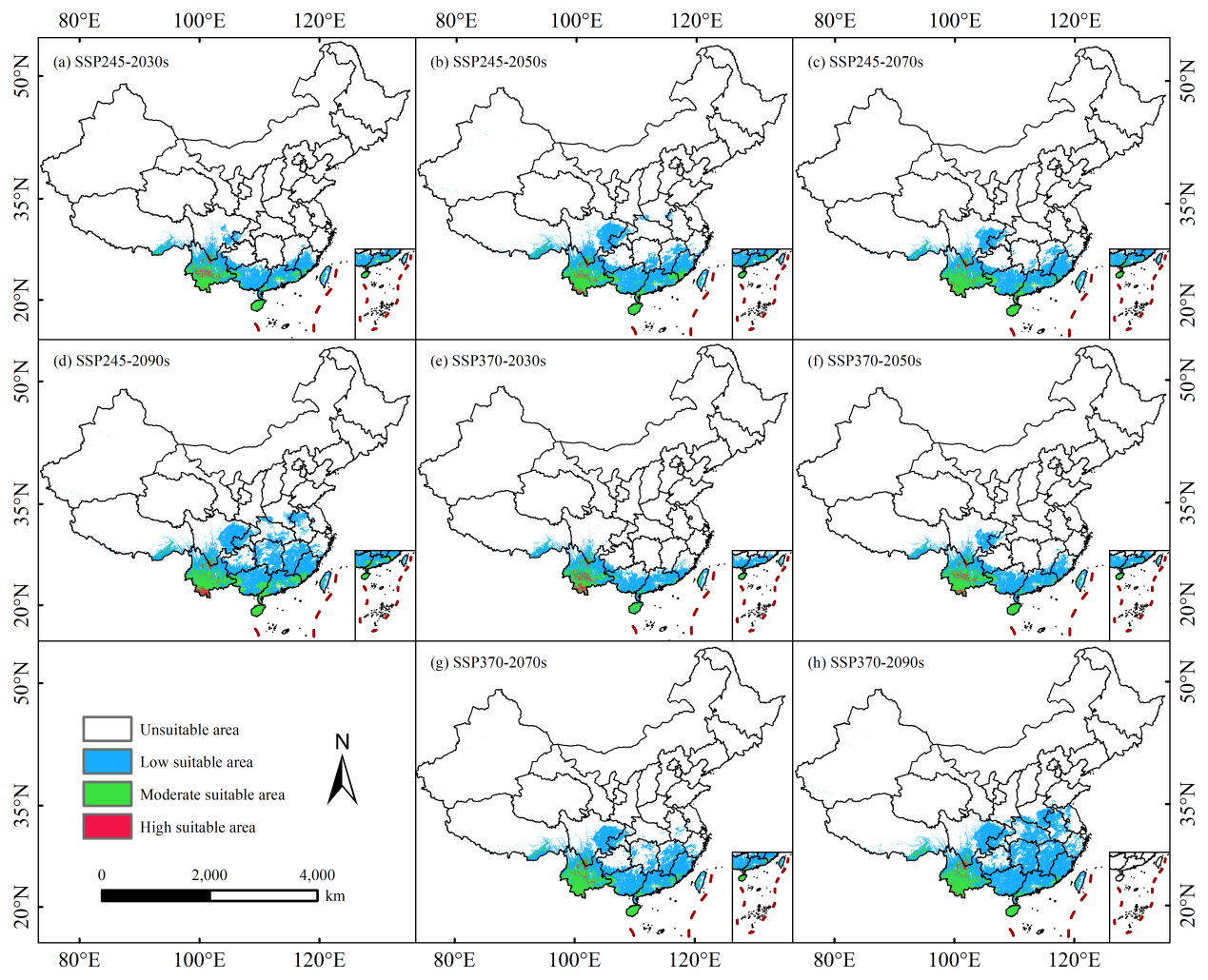


Figure S1 Predicted potential suitable habitats for *C. arabica* in China under different climate scenarios (a-h).


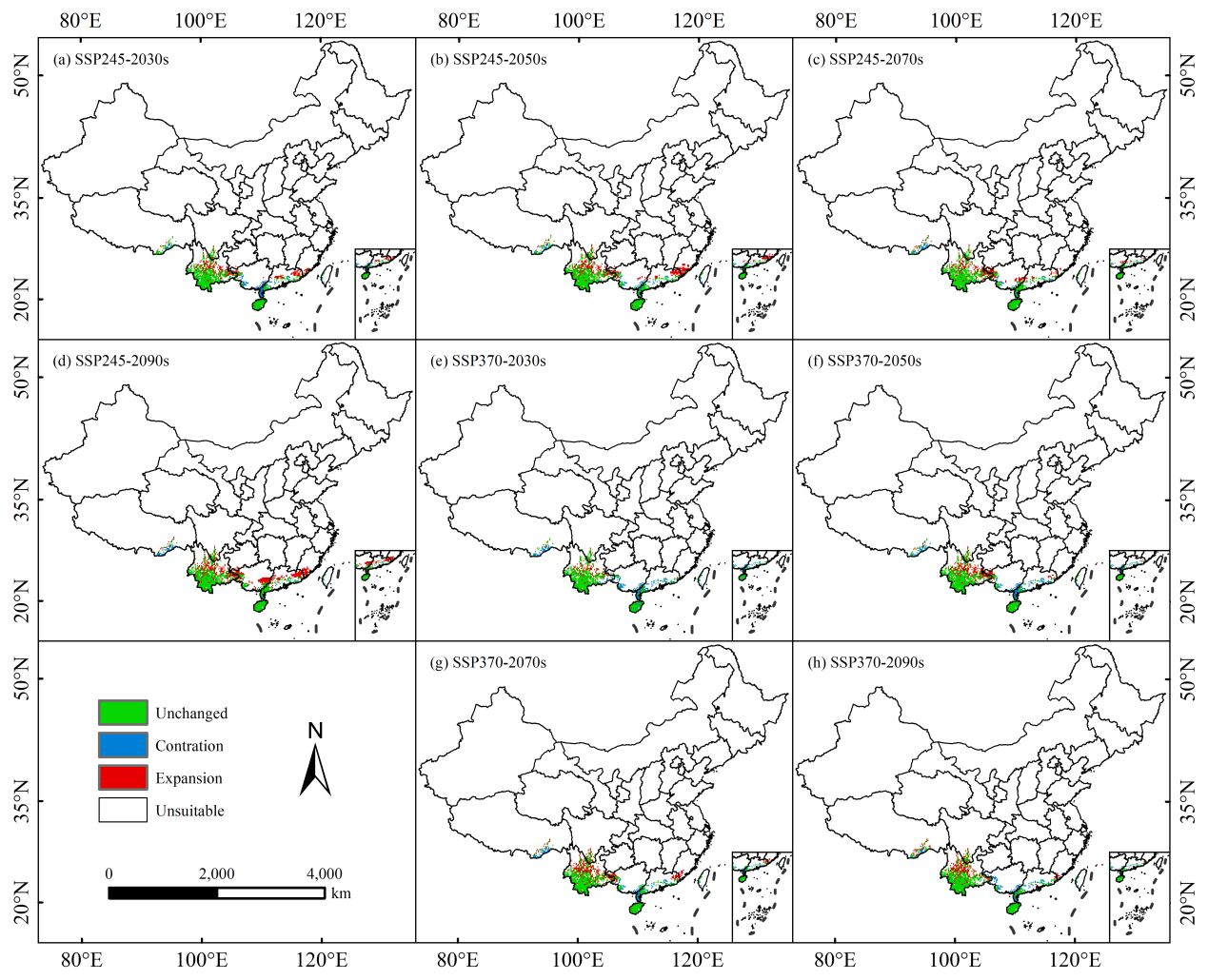


Figure S2 Spatial variations in the moderate and high suitable for *C. arabica* in China under different climate scenarios (a-h).


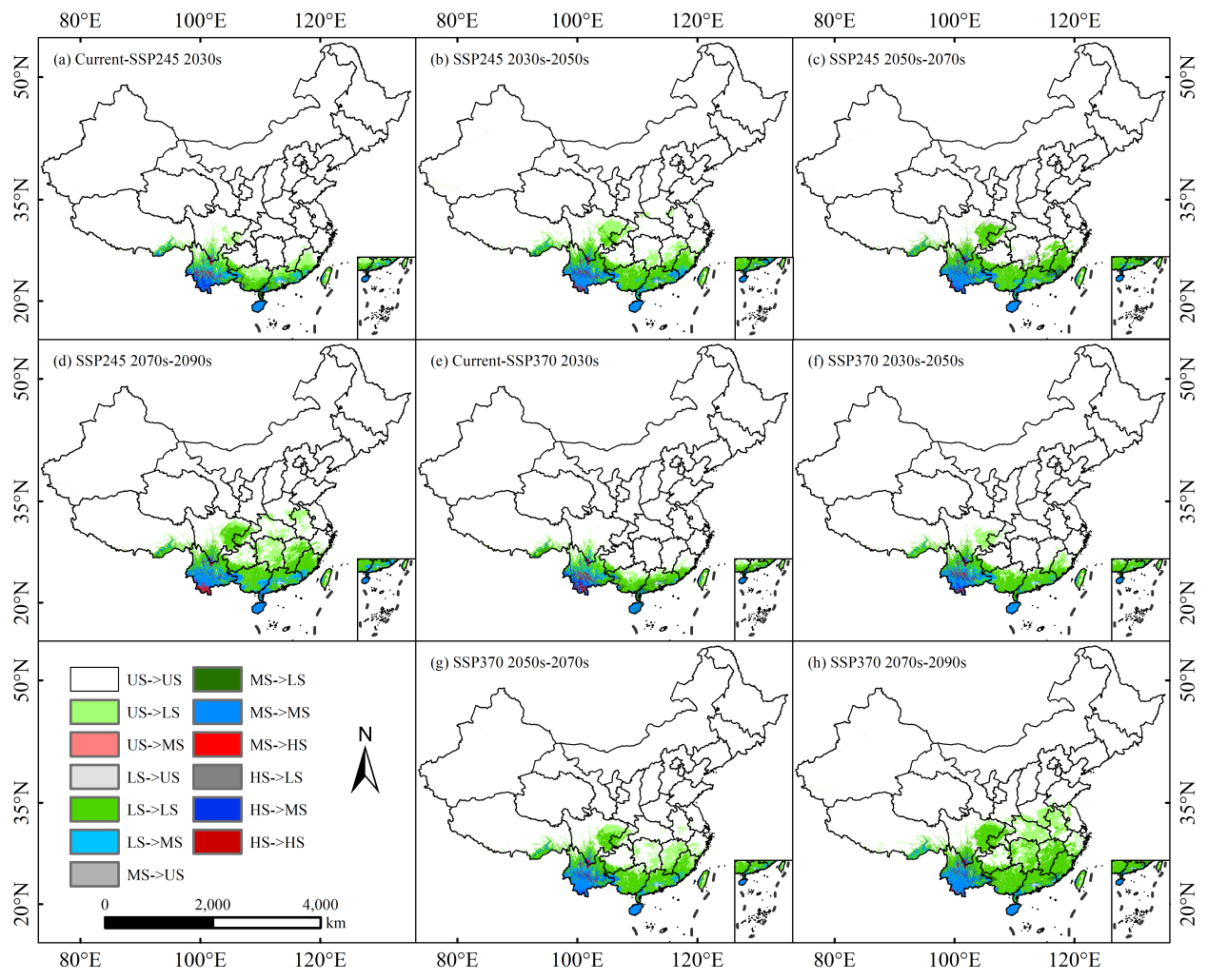


Figure S3 Different suitable habitats transfer of *C. arabica* in China under future climate scenarios (a-h).


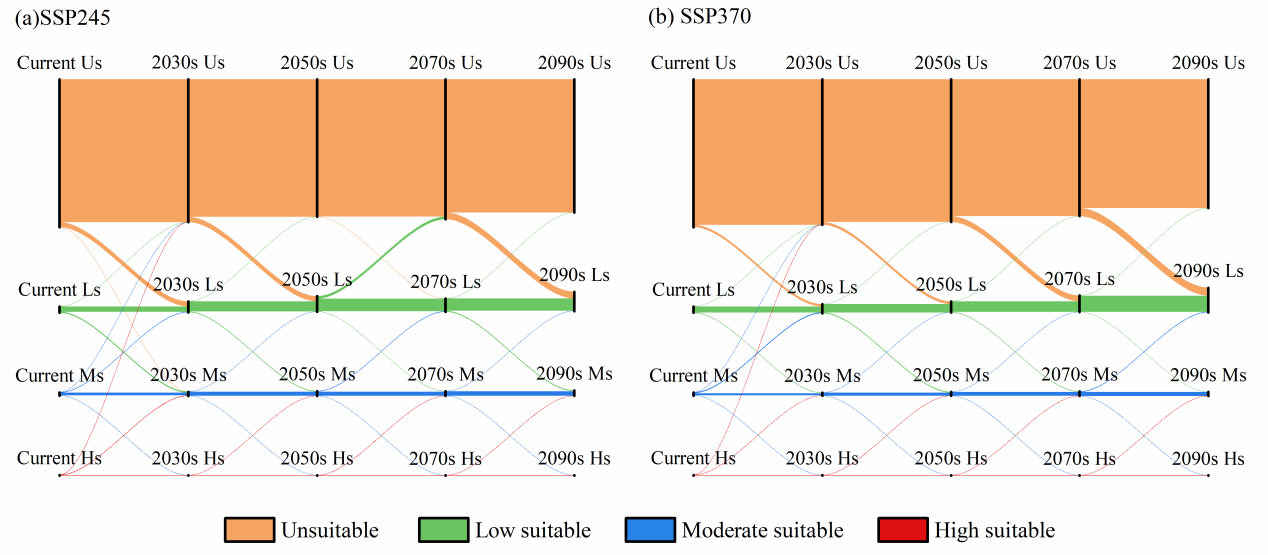


Figure S4 Area transfer of *C. arabica* in China under future climate scenarios. (a) Area transition under the SSP245 scenario; (b) Area transition under the SSP370 scenario.


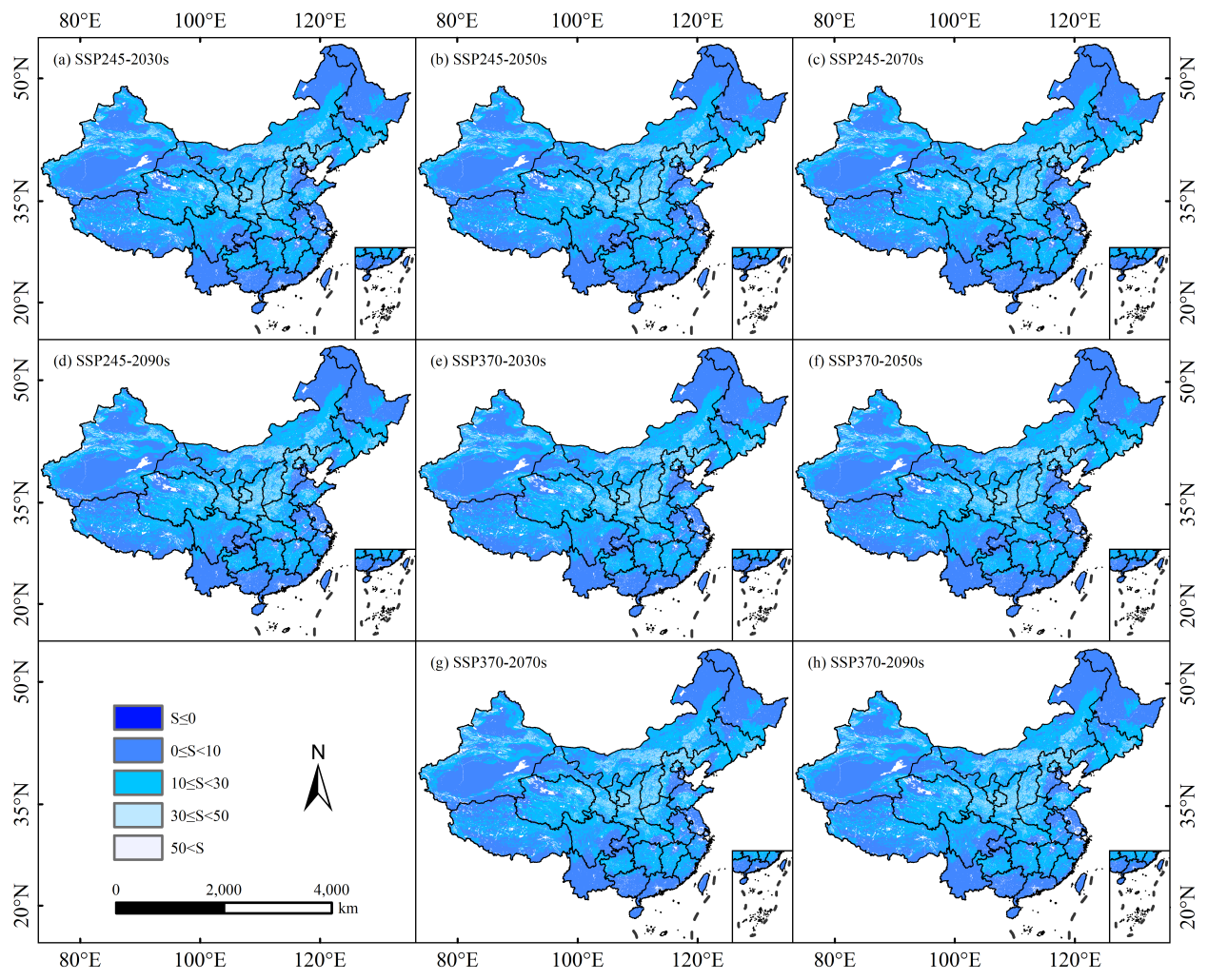


Figure S5 Multivariate environmental similarity surface variable analysis for *C. arabica* in China under different combinations of climate change scenarios (a-h).


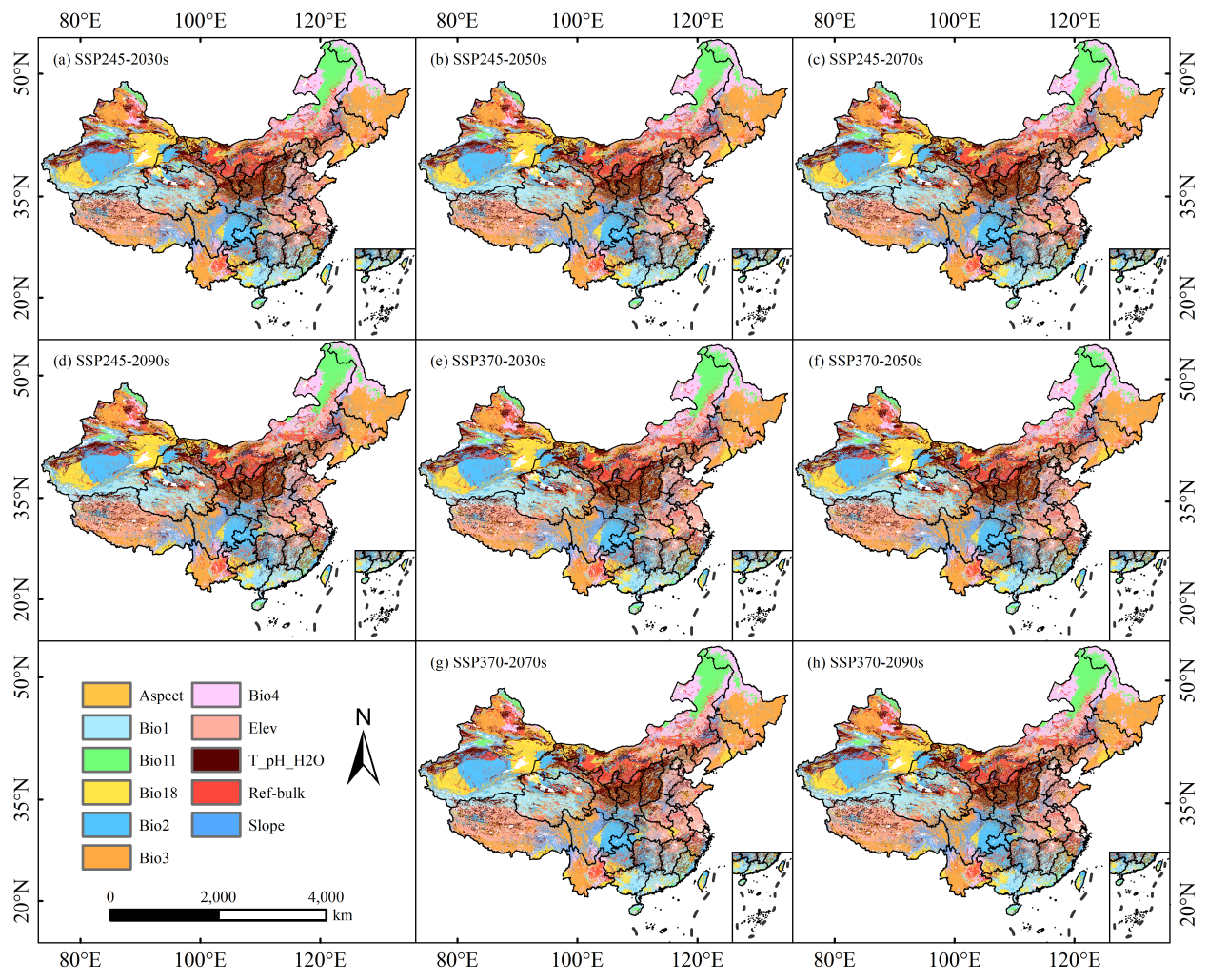


Figure S6 Most dissimilar variable analysis for *C. arabica* in China under different combinations of climate change scenarios (a-h).


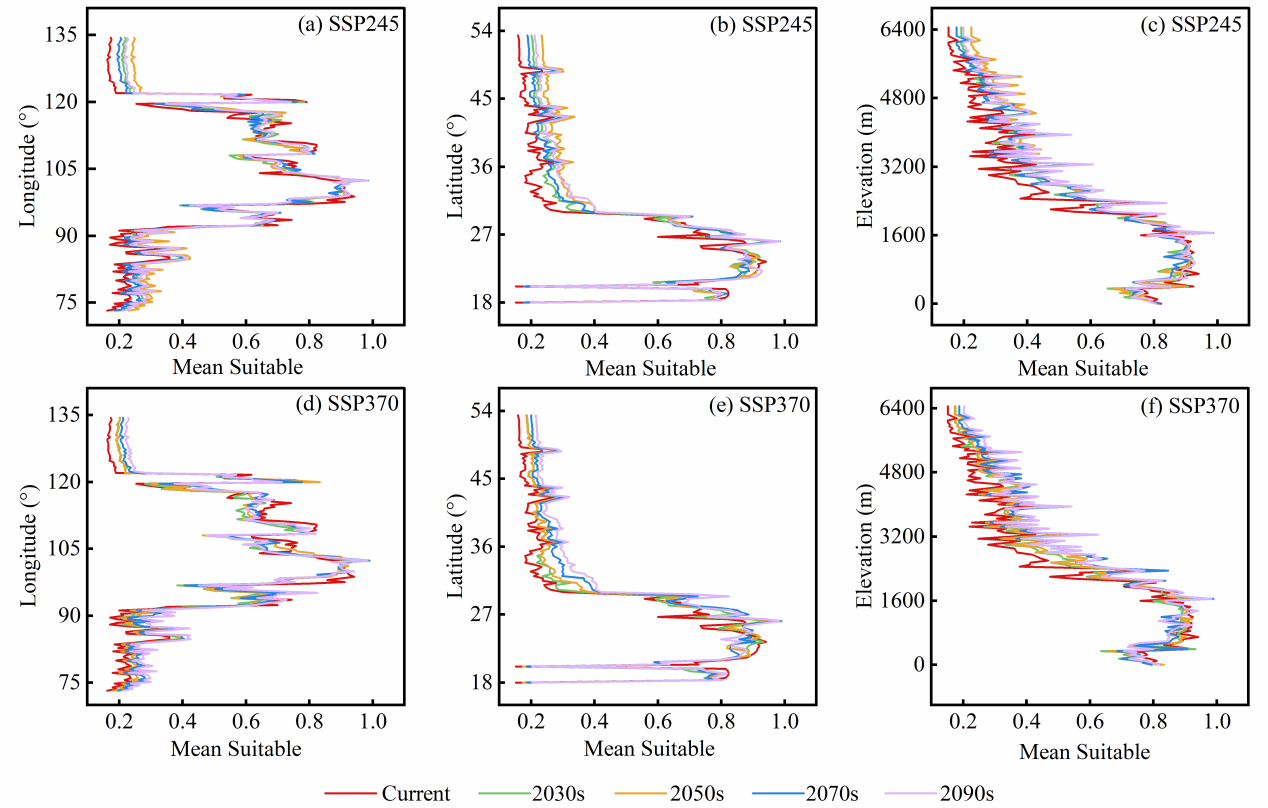


Figure S7 Variation of latitude, longitude and elevation of *C. arabica* in China under different combinations of climate change scenarios (a-f).
