# Supplementary material for: Climate change provides opportunities for the cultivation of Coffea arabica in China, an integrated model analysis based on biomod2
Source: Front Plant Sci. 2026 Mar 20;17:1806108. doi: 10.3389/fpls.2026.1806108 (PMC13047709; doi:10.3389/fpls.2026.1806108)
Supplement: Supplementary Figure S1 — Predicted potential suitable habitats for C. arabica in China under different climate scenarios (a–h). [file DataSheet1.zip › Supplementary file/References.docx]

Allan, R. P., Arias, P. A., Berger, S., Canadell, J. G., Cassou, C., Chen, D., ... & Zickfeld, K. (2023). Intergovernmental panel on climate change (IPCC). Summary for policymakers. In Climate change 2021: *The physical science basis. Contribution of working group I to the sixth assessment report of the intergovernmental panel on climate change* (pp. 3-32). Cambridge University Press.

Ambrósio, G., Doelman, J. C., Schipper, A. M., Stehfest, E., van Vuuren, D., & Harfoot, M. et al. (2024). Global sustainability scenarios lead to regionally different outcomes for terrestrial biodiversity. *Environ. Res. Lett.,* 19, 104055. <https://doi.org/10.1088/1748-9326/ad73eb>

Bellard, C., Bertelsmeier, C., Leadley, P., Thuiller, W., & Courchamp, F. (2012). Impacts of climate change on the future of biodiversity. *Ecology letters*, 15(4), 365-377. <https://doi.org/10.1111/j.1461-0248.2011.01736.x>

Bunn, C., Läderach, P., Ovalle Rivera, O., & Kirschke, D. (2015). A bitter cup: climate change profile of global production of Arabica and Robusta coffee. *Climatic change*, 129(1), 89-101. doi: 10.1007/s10584-014-1306-x

Byrareddy, V. M., Kath, J., Kouadio, L., Mushtaq, S., & Geethalakshmi, V. (2024). Assessing scale-dependency of climate risks in coffee-based agroforestry systems. *Scientific Reports*, 14(1), 8028. doi: 10.1038/s41598-024-58790-5

Cai, L., Kreft, H., Taylor, A., Zhang, J., Feng, X., & Svenning, J.-C. et al. (2023). Global models and predictions of plant diversity based on advanced machine learning techniques. *New Phytol.,* 237, 1432–1445. https://doi.org/10.1111/nph.18533

Chen, I. C., Hill, J. K., Ohlemüller, R., Roy, D. B., & Thomas, C. D. (2011). Rapid range shifts of species associated with high levels of climate warming. *Science,* 333(6045), 1024-1026. doi: 10.1126/science.1206432

Chen, J., Zhang, Y., Liu, W., Wang, C., Ma, F., & Xu, H. (2023). Distribution patterns and determinants of invasive alien plants in China. *Plants* 12, 2341. doi: 10.3390/plants12122341

Davis, A. P., Chadburn, H., Moat, J., O’Sullivan, R., Hargreaves, S., & Nic Lughadha, E. (2019). High extinction risk for wild coffee species and implications for coffee sector sustainability. *Sci. Adv*. 5, eaav3473. doi: 10.1126/sciadv.aav3473

Davis, A. P., Gole, T. W., Baena, S., & Moat, J. (2012). The impact of climate change on indigenous arabica coffee (*Coffea arabica*): predicting future trends and identifying priorities. *PloS One* 7, e47981. doi: 10.1371/journal.pone.0047981

Dormann, C. F., Calabrese, J. M., Guillera‐Arroita, G., Matechou, E., Bahn, V., Bartoń, K., ... & Hartig, F. (2018). Model averaging in ecology: A review of Bayesian, information-theoretic, and tactical approaches for predictive inference. *Ecol. Monogr.* 88, 485–504. doi: 10.1002/ecm.1309

Fang, Y., Zhang, X., Wei, H., Wang, D., Chen, R., Wang, L., & Gu, W. (2021). Predicting the invasive trend of exotic plants in China based on the ensemble model under climate change: A case for three invasive plants of *Asteraceae*. *Sci. Total Environ. 756,* 143841. doi: 10.1016/j.scitotenv.2020.143841

Gan, T., Liu, Q., Xu, D., He, Z., & Zhuo, Z. (2025). Assessing the impact of climate change on Hippophae neurocarpa in China using biomod2 modeling. *Agriculture 15,* 722. doi: 10.3390/agriculture15070722

Gebrewahid, Y., Abrehe, S., Meresa, E., Eyasu, G., Abay, K., Gebreab, G., ... & Darcha, G. (2020). Current and future predicting potential areas of *Oxytenanthera abyssinica* (A. Richard) using MaxEnt model under climate change in Northern Ethiopia. *Ecol. Processes 9,* 1–15. doi: 10.1186/s13717-019-0210-8

Gilani, H., Goheer, M. A., Ahmad, H., & Hussain, K. (2020). Under predicted climate change: Distribution and ecological niche modelling of six native tree species in Gilgit-Baltistan, Pakistan. *Ecol. Indic.* 111, 106049. doi: 10.1016/j.ecolind.2019.106049

Gong, X., Chen, Y., Wang, T., Jiang, X., Hu, X., & Feng, J. (2020). Double-edged effects of climate change on plant invasions: Ecological niche modeling global distributions of two invasive alien plants. *Sci. Total Environ.* 740, 139933. doi: 10.1016/j.scitotenv.2020.139933

Hao, T., Elith, J., Guillera‐Arroita, G., & Lahoz‐Monfort, J. J. (2019). A review of evidence about use and performance of species distribution modelling ensembles like BIOMOD. *Diversity Distrib.* 25, 839–852. doi: 10.1111/ddi.12892

Hasegawa, T., Sakurai, G., Fujimori, S., Takahashi, K., Hijioka, Y., & Masui, T. (2021). Extreme climate events increase risk of global food insecurity and adaptation needs. *Nat. Food 2*, 587–595. doi: 10.1038/s43016-021-00335-4

Huang, D., An, Q., Huang, S., Tan, G., Quan, H., Chen, Y., ... & Liao, H. (2023). Biomod2 modeling for predicting the potential ecological distribution of three *Fritillaria* species under climate change. *Sci. Rep. 13*, 18801. doi: 10.1038/s41598-023-45887-6

Kakpo, S. B., Aoudji, A. K. N., Gnanguènon-Guéssè, D., Gbètoho, A. J., Koura, K., Djotan, G. K., & Ganglo, J. C. (2021). Spatial distribution and impacts of climate change on *Milicia excelsa* in Benin, West Africa. *J. Forest. Res.* 32, 143–150. doi: 10.1007/s11676-019-01069-7

Kass, J. M., Muscarella, R., Galante, P. J., Bohl, C. L., Pinilla‐Buitrago, G. E., Boria, R. A., ... & Anderson, R. P. (2021). ENMeval 2.0: Redesigned for customizable and reproducible modeling of species’ niches and distributions. *Methods Ecol. Evol.* 12, 1602–1608. doi: 10.1111/2041-210X.13628

Kong, L. D., Huang, M. S., Liu, C., Wang, S. Q., Liu, D. Y., Wang, R. H. (2025). Analysis of suitable habitat evolution for Northern Magnoliaceae plants under climate change scenarios. *J. Ecol.* 19), 1–14. doi:10.20103/j.stxb.202501160149.

Läderach, P., Ramirez–Villegas, J., Navarro-Racines, C., Zelaya, C., Martinez–Valle, A., & Jarvis, A. (2017). Climate change adaptation of coffee production in space and time. *Clim. Change* 141, 47–62. doi: 10.1007/s10584-016-1788-9

Latimer, A. M., Wu, S., Gelfand, A. E., & Silander Jr, J. A. (2006). Building statistical models to analyze species distributions. *Ecol. Appl.* 16, 33–50. doi: 10.1890/04-0609

Li, X., Wang, Z., Wang, S., & Qian, Z. (2024). MaxEnt and Marxan modeling to predict the potential habitat and priority planting areas of *Coffea arabica* in Yunnan, China under climate change scenario. *Front. Plant Sci.* 15, 1471653. doi: 10.3389/fpls.2024.1471653

Liu, Y., Huang, P., Lin, F., Yang, W., Gaisberger, H., Christopher, K., & Zheng, Y. (2019). MaxEnt modelling for predicting the potential distribution of a near threatened rosewood species (Dalbergia cultrata Graham ex Benth). *Ecol. Eng.* 141, 105612. doi: 10.1016/j.ecoleng.2019.105612

Muscarella, R., Galante, P. J., Soley‐Guardia, M., Boria, R. A., Kass, J. M., Uriarte, M., & Anderson, R. P. (2014). ENM eval: An R package for conducting spatially independent evaluations and estimating optimal model complexity for Maxent ecological niche models. *Methods Ecol. Evol.* 5, 1198–1205. doi: 10.1111/2041-210X.12261

Pais, S., Aquilué, N., Campos, J., Sil, Â., Marcos, B., Martínez-Freiría, F., ... & Regos, A. (2020). Mountain farmland protection and firesmart management jointly reduce fire hazard and enhance biodiversity and carbon sequestration. *Ecosyst. Serv.* 44, 101143. doi: 10.1016/j.ecoser.2020.101143

Phillips, S. J., Anderson, R. P., Dudík, M., Schapire, R. E., & Blair, M. E. (2017). Opening the black box: An opensource release of Maxent. *Ecography* 40, 887–893. doi: 10.1111/ecog.03049

Pio, D. V., Engler, R., Linder, H. P., Monadjem, A., Cotterill, F. P., Taylor, P. J., ... & Guisan, A. (2014). Climate change effects on animal and plant phylogenetic diversity in southern Africa. *Global Change Biol.* 20, 1538–1549. doi: 10.1111/gcb.12524

Pramanik, M., Paudel, U., Mondal, B., Chakraborti, S., & Deb, P. (2018). Predicting climate change impacts on the distribution of the threatened *Garcinia indica* in the Western Ghats, India. *Climate Risk Manage.* 19, 94–105. doi: 10.1016/j.crm.2017.11.002

Tavares, P. D. S., Giarolla, A., Chou, S. C., Silva, A. J. D. P., & Lyra, A. D. A. (2018). Climate change impact on the potential yield of Arabica coffee in southeast Brazil. *Regional Environ. Change* 18, 873–883. doi: 10.1007/s10113-017-1236-z

Thuiller, W., Lafourcade, B., Engler, R., & Araújo, M. B. (2009). BIOMOD–a platform for ensemble forecasting of species distributions. *Ecography 32*, 369–373. doi: 10.1111/j.1600-0587.2008.05742.x

Tun, W., Yoon, J., Jeon, J. S., & An, G. (2021). Influence of climate change on flowering time. *J. Plant Biol.* 64, 193–203. doi: 10.1007/s12374-021-09300-x

Wang, C., Liu, C., Wan, J., & Zhang, Z. (2016). Climate change may threaten habitat suitability of threatened plant species within Chinese nature reserves. *PeerJ* 4, e2091. doi: 10.7717/peerj.2091

Wang, Y., Liu, H., Xu, J., Yu, S., Huang, Y., Zhang, Y., ... & Chen, W. (2023). Prediction of suitable planting areas of *Rubia cordifolia* in China based on a species distribution model and analysis of specific secondary metabolites. *Ind. Crops Prod.* 206, 117651. doi: 10.1016/j.indcrop.2023.117651

Wang, X., Wang, Y., Hu, G., Hong, D., Guo, T., Li, J., ... & Qiu, M. (2022). Review on factors affecting coffee volatiles: From seed to cup. *J. Sci. Food Agric.* 102, 1341–1352. doi: 10.1002/jsfa.11647

Wang, T., Zhang, T., An, W., Wang, Z., & Li, C. (2024). Predicting the potential geographic distribution of invasive freshwater apple snail *Pomacea canaliculate* (Lamarck, 1819) under Climate change based on Biomod2. *Agronomy* 14, 650. doi: 10.3390/agronomy14040650

Wen, J., Chen, C., Liu, Q., Zhang, T., Gu, Y., Han, Y., ... & He, Y. (2025). Environmental drivers of *Leonurus japonicus* habitat suitability: An ensemble model approach. *Ind. Crops Prod*. 234, 121562. doi: 10.1016/j.indcrop.2025.121562

Wiens, J. A., Stralberg, D., Jongsomjit, D., Howell, C. A., & Snyder, M. A. (2009). Niches, models, and climate change: assessing the assumptions and uncertainties. *Proc. Natl. Acad. Sci.* 106, 19729–19736. doi: 10.1073/pnas.0901639106

Xu, Y., Huang, Y., Zhao, H., Yang, M., Zhuang, Y., & Ye, X. (2021). Modelling the effects of climate change on the distribution of endangered *Cypripedium japonicum* in China. *Forests* 12, 429. doi: 10.3390/f12040429

Zhang, S., Liu, X., Li, R., Wang, X., Cheng, J., Yang, Q., & Kong, H. (2021). AHP-GIS and MaxEnt for delineation of potential distribution of Arabica coffee plantation under future climate in Yunnan, China. *Ecol. Indic*. 132, 108339. doi: 10.1016/j.ecolind.2021.108339

Zhang, Q., Shen, X., Jiang, X., Fan, T., Liang, X., & Yan, W. (2023). MaxEnt modeling for predicting suitable habitat for endangered tree *Keteleeria davidiana* (Pinaceae) in China. *Forests* 14, 394. doi: 10.3390/f14020394

Zhang, H., Zhang, X., Zhang, G., Sun, X., Chen, S., & Huang, L. (2024). Assessing the quality ecology of endemic tree species in China based on machine learning models and UPLC methods: The example of *Eucommia ulmoides Oliv*. *J. Clean. Prod*. 452, 142021. doi: 10.1016/j.jclepro.2024.142021

Zhao, G., Cui, X., Sun, J., Li, T., Wang, Q. I., Ye, X., & Fan, B. (2021). Analysis of the distribution pattern of Chinese *Ziziphus jujuba* under climate change based on optimized biomod2 and MaxEnt models. *Ecol. Indic.* 132, 108256. doi: 10.1016/j.ecolind.2021.108256

Zhao, Y., Zhang, Y., Yan, Y., Wen, Y., & Zhang, D. (2024). Geographic distribution and impacts of climate change on the suitable habitats of two *alpine Rhododendron* in Southwest China. *Global Ecol. Conserv.* 54, e03176. doi: 10.1016/j.gecco.2024.e03176

Zhu, Y., Dou, X., Wang, R., Xie, M. E., Huang, W., & Li, M. (2021). Climate change impact on the region suitable for *Coffea arabica* growth in Yunnan province. *Acta Meteorol. Sin.* 79, 878–887.
